# Prophylactic Prednisolone Promotes AAV5 Hepatocyte Transduction Through the Novel Mechanism of AAV5 Coreceptor Platelet-Derived Growth Factor Receptor Alpha Upregulation and Innate Immune Suppression

**DOI:** 10.1089/hum.2023.065

**Published:** 2024-01-16

**Authors:** Britta Handyside, Lening Zhang, Bridget Yates, Lin Xie, Ashrafali Mohamed Ismail, Ryan Murphy, Brian Baridon, Cheng Su, Taren Bouwman, Linley Mangini, Jorden Tahquechi, Sandra Salcido, Wesley C. Minto, William T. Keenan, Ioanna Ntai, Choong-Ryoul Sihn, Sherry Bullens, Stuart Bunting, Sylvia Fong

**Affiliations:** ^1^ Biology Research; BioMarin Pharmaceutical, Inc.; Novato, California, USA.; ^2^ Global Clinical Sciences; BioMarin Pharmaceutical, Inc.; Novato, California, USA.; ^3^ Translational Sciences; BioMarin Pharmaceutical, Inc.; Novato, California, USA.

**Keywords:** AAV, valoctocogene roxaparvovec, AAV5-hFVIII-SQ, AAV transduction, PDGFRα, prophylactic corticosteroids

## Abstract

Adeno-associated virus (AAV) vectors are used to deliver therapeutic transgenes, but host immune responses may interfere with transduction and transgene expression. We evaluated prophylactic corticosteroid treatment on AAV5-mediated expression in liver tissue. Wild-type C57BL/6 mice received 6 × 10^13^ vg/kg AAV5-HLP-hA1AT, an AAV5 vector carrying a human α1-antitrypsin (hA1AT) gene with a hepatocyte-specific promoter. Mice received 4 weeks of daily 2 mg/kg prednisolone or water starting day −1 or 0 before vector dosing. Mice that received prophylactic corticosteroids had significantly higher serum hA1AT protein than mice that did not, starting at 6 weeks and persisting to the study end at 12 weeks, potentially through a decrease in the number of low responders. RNAseq and proteomic analyses investigating mechanisms mediating the improvement of transgene expression found that prophylactic corticosteroid treatment upregulated the AAV5 coreceptor platelet-derived growth factor receptor alpha (PDGFRα) on hepatocytes and downregulated its competitive ligand PDGFα, thus increasing the uptake of AAV5 vectors. Evidently, prophylactic corticosteroid treatment also suppressed acute immune responses to AAV. Together, these mechanisms resulted in increased uptake and preservation of the transgene, allowing more vector genomes to be available to assemble into stable, full-length structures mediating long-term transgene expression. Prophylactic corticosteroids represent a potential actionable strategy to improve AAV5-mediated transgene expression and decrease intersubject variability.

## INTRODUCTION

Adeno-associated virus (AAV) vectors efficiently transduce human cells but also stimulate innate and adaptive immune responses against the AAV capsid, transgene product, or AAV-transduced cells.^1–4^ Host immune responses may hamper transduction and contribute to interparticipant variability in clinical trials.^1,2,5–11^ Host cell pattern recognition receptors induce proinflammatory cytokines, and antiviral responses lead to vector deoxyribonucleic acid (DNA) degradation.^5,12^ Adaptive immune responses, including expansion of CD8^+^ T cells directed against transgene-expressing target cells, cause inflammation and damage.^7,13,14^

Corticosteroids inhibit immune responses by suppressing transcription of proinflammatory cytokines and chemokines.^15,16^ Corticosteroids are frequently used with AAV gene therapies to treat alanine aminotransferase (ALT) elevations in liver-targeted gene therapies, and preserve transgene expression in some cases.^8,10^ Differential timing of corticosteroid use across trials of the gene therapy valoctocogene roxaparvovec (AAV5-hFVIII-SQ) and differences in responses suggest that earlier corticosteroid use may promote transgene expression.^8,9,17–19^

Prophylactic corticosteroids administered before AAV dosing may promote transgene expression. In mice, prophylactic dexamethasone administered 2 h before AAV9 dosing increased hepatic levels of vector genomes.^20^ Nonhuman primates (NHPs) treated with prophylactic prednisolone before AAV dosing had less CD8^+^ T cell infiltration and apoptosis than those who did not.^21^ However, prednisolone treatment in mice 1 week after AAV5-hFVIII-SQ dosing did not affect transgene expression.^22^ Similarly, dexamethasone treatment initiated 1 year after AAV9 did not change transgene expression levels in dogs.^20^

In this study, we assessed transgene expression in mice after prophylactic prednisolone before AAV5-mediated gene therapy over 12 weeks. After confirming prophylactic prednisolone increased transgene expression, we investigated potential mechanisms of action at 2 and 24 h post-AAV dosing using RNAseq, proteomics, and targeted molecular analyses. Not only did prophylactic prednisolone suppress innate immune responses, it also upregulated the expression of the AAV5 coreceptor platelet-derived growth factor receptor alpha (PDGFRα).

## MATERIALS AND METHODS

### Vector constructs

Valoctocogene roxaparvovec (AAV5-hFVIII-SQ) gene therapy for severe hemophilia A is a recombinant AAV5 vector containing a human B-domain–deleted FVIII cDNA (hFVIII-SQ) controlled by a hepatocyte-selective promoter.^8,23,24^ Mouse studies with serial blood sampling used the reporter vector AAV5-HLP-hA1AT, which delivers a human α1-antitrypsin (hA1AT) gene, instead of AAV5-hFVIII-SQ because hA1AT protein is nonimmunogenic in mice, and serial blood draws activate the clotting cascade and consume factor VIII (FVIII), introducing variability.^25,26^

### Study design

**Figure 1.**
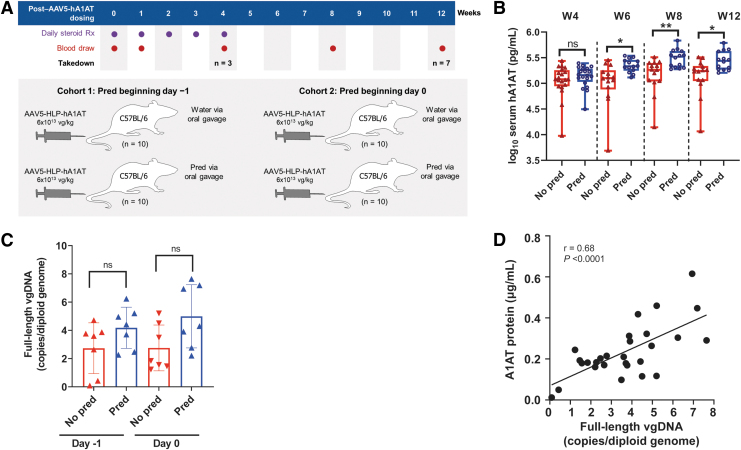
Effect of prophylactic prednisolone treatment over 12 weeks. **(A)** Design of the 12-week study. **(B)** Variation in serum hA1AT levels across 12 weeks from mice dosed with 6 × 10^13^ vg/kg AAV5-HLP-hA1AT. **(C)** Levels of full-length vector DNA in the liver at week 12 (*n* = 7) by ddPCR. **(D)** Pearson correlation between levels of full-length vector DNA in the liver versus serum A1AT protein at week 12 (*n* = 28). *p < 0.05; **p < 0.01. Liver samples were collected at takedown. In **(B)**, data are median, Q1, Q3, minimum, and maximum. Bar graphs show mean ± SD. Symbols are individual data points. In **(C)**, each bar represents average of three data points. “No pred” includes mice that received control 2 or 24 h before AAV; “pred” includes mice that received prednisolone at day −1 or 0 before AAV. In **(B, C)**, a Student's *t*-test was performed. A1AT, α1-antitrypsin; AAV, adeno-associated virus; AAV5-HLP-hA1AT, AAV serotype 5 hybrid liver promoter human α1-antitrypsin; ddPCR, droplet digital polymerase chain reaction; DNA, deoxyribonucleic acid; ns, not significant; pred, prednisolone; Q1, first quartile; Q3, third quartile; SD, standard deviation; vg, vector genome.

We assessed the effects of prophylactic steroids over 12 weeks with wild-type C57BL/6 mice (Jackson Laboratory, Bar Harbor, ME) that received either water or 2 mg/kg prednisolone in water vehicle by oral gavage beginning on day −1 or 0 before AAV dosing and continuing for 4 weeks (Fig. 1A). Blood was drawn at baseline and weeks 1, 4, 6, 8, and 12 postdose for serum hA1AT measurement; takedown cohorts were collected at weeks 4 (*n* = 3) and 12 (*n* = 7) and livers were collected for vector genome quantification. Mice were dosed with 6 × 10^13^ vg/kg AAV5-HLP-hA1AT. The impact of prophylactic methylprednisolone and rituximab before dosing with 6 × 10^13^ vg/kg AAV5-hFVIII-SQ was assessed in NHPs (Supplementary Methods section in Supplementary Data).

**Figure 2.**
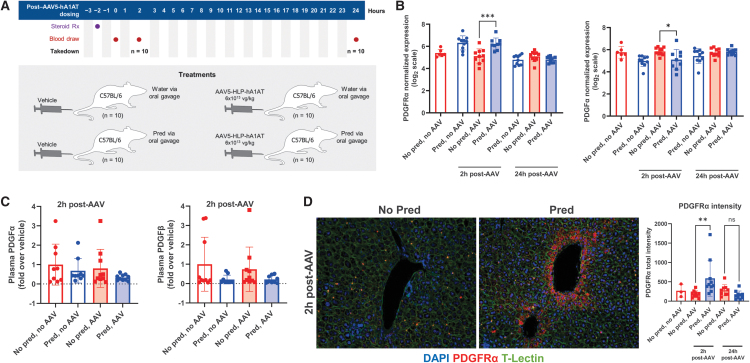
Effect of prophylactic prednisolone treatment on PDGFRα receptor and ligands at 2 and 24 h after vector dosing. **(A)** Study design of the 2- and 24-h study. **(B)** Normalized expression of PDGFRα and ligand PDGFα in liver homogenate by RNAseq. **(C)** Plasma PDGFα and PDGFβ protein levels assessed by ELISA and Olink assay, respectively. **(D)** PDGFRα protein levels in hepatocytes following AAV5-HLP-hA1AT administration with and without prophylactic prednisolone treatment. *p < 0.05; **p < 0.01; ***p < 0.001. Bar graphs show mean ± SD; *symbols* are individual data points. AAV denotes AAV5-HLP-hA1AT dosing. In **(D)**, images are at 20 × magnification. For **(B)**, a Welch's unpaired *t*-test was performed. For plasma PDGFRα in **(C)** a one-way ANOVA followed by Tukey's multiple comparison showed no significant differences. For plasma PDGFRβ in **(C)** Olink analysis using Welch's correction did not show any significance. For **(D)**, a one-way ANOVA followed by Tukey's multiple comparison was performed. ANOVA, analysis of variance; DAPI, 4′,6-diamidino-2-phenylindole fluorescent stain; ELISA, enzyme-linked immunosorbent assay; ns, not significant; PDGF, platelet-derived growth factor; PDGFR, platelet-derived growth factor receptor.

We also assessed the effects of prophylactic steroids on events immediately after AAV dosing with wild-type C57BL/6 mice who received one of four treatments (prophylactic prednisolone or water and 6 × 10^13^ vg/kg AAV5-HLP-hA1AT or vehicle) before euthanization at 2 or 24 h post-AAV dose (Fig. 2A). Blood samples were taken predose and terminally for hA1AT and other protein assays. Livers were collected for RNAseq, protein biomarker detection using an Olink assay, *N*-glycome analyses, and immunohistochemistry.

In addition, we assessed how natural PDGFRα variation correlates with transgene expression by dosing wild-type C57BL/6 and BalbC (Jackson Laboratory) mice with 6 × 10^13^ AAV5-hFVIII-SQ. Mice were euthanized at 5 weeks postdose, and blood and liver samples were collected for assessment of FVIII-SQ protein, transgene DNA, and PDGFRα protein.

### Ethics statement

Mouse protocols were approved by the Animal Resource Committee of BioMarin Pharmaceutical, Inc. and the Institutional Animal Care and Use Committee of the Buck Institute. NHP protocols were approved by the Institutional Animal Care and Use Committee at the Charles River Laboratory (San Francisco, CA).

### RNAseq and pathway enrichment analyses

mRNA samples were prepared using Illumina Stranded mRNA kits (Illumina, San Diego, CA) and sequenced on a NovaSeq 6000 v1.5 using single-end 100-bp reads. Reads were analyzed and mapped to the GRCm38/mm10 mouse genome assembly.^27^ Differential expression was determined using the edgeR package in R (R Foundation, Vienna, Austria). Gene set enrichment analysis was performed using hallmark gene sets from MSigDb in R, using normalized enrichment score (NES) and Benjamini–Hochberg-adjusted *p* value.^28,29^

### Protein assays

hA1AT protein in mice was measured in serum (12-week study) or plasma (24-h study) using an enzyme-linked immunosorbent assay (ELISA) detecting hA1AT without mouse cross-reactivity (Human Serpin A1 DuoSet ELISA, DY1268; R&D Systems, Minneapolis, MN; Supplementary Methods section in Supplementary Data).

hFVIII-SQ protein was measured in plasma samples using a sandwich ELISA utilizing human-specific anti-FVIII capture (Green Mountain Antibodies, Burlington, VT) and detection (F8C-EIA; Affinity Biologics, Ancaster, ON, Canada) antibody pairs, as previously described.^23^

Complement system activity was assessed with plasma C3b (Supplementary Methods section in Supplementary Data). An Olink proteomic assay was used to explore protein expression (Supplementary Methods section in Supplementary Data). Plasma PDGFα/α was measured using a Mice PDGFα/α ELISA kit (Abcam, Cambridge, United Kingdom).

### Plasma ALT

Mouse plasma ALT levels were assessed at 2 and 24 h post-AAV dose and over 12 weeks (Supplementary Methods section in Supplementary Data).

### DNA *in situ* hybridization

Hepatocytes staining positive for vector genome and *in situ* hybridization (ISH) area per cell were quantified in formalin-fixed paraffin-embedded (FFPE) liver sections of 5 μm prepared as described.^23^ One whole liver section was acquired per animal, and two regions were randomly selected for analysis using Visiopharm (Hørsholm, Denmark).

### Droplet digital polymerase chain reaction

Vector genome quantification, including full-length genomes and overall total genomes, was performed with drop-phase droplet digital polymerase chain reaction (ddPCR) analyses targeting the AAV5-hA1AT-HLP transgene sequence, as described^23^ (Supplementary Methods section in Supplementary Data), following DNA and ribonucleic acid (RNA) extraction using the AllPrep DNA/RNA Mini kit (Qiagen, Hilden, Germany).

### *N*-glycome analysis

Changes in *N*-glycosylation in mouse livers at 2 and 24 h post-AAV dose were assessed (Supplementary Methods section in Supplementary Data).

### Immunohistochemistry

Hepatic expression and distribution of PDGFRα and viral protein 3 (VP3) were measured by immunohistochemistry. FFPE livers sectioned at 5-μm thickness were collected on Leica Microsystems Plus Slides (Leica Biosystems, Buffalo Grove, IL). Immunostaining was performed using a Leica BOND RX Autostainer (Supplementary Methods section in Supplementary Data).

### hFVIII-SQ vector genome quantification

AAV5-hFVIII-SQ vector genome levels were measured with a quantitative real-time PCR assay using a TaqMan DNA probe and primers specific to hFVIII-SQ after genomic liver DNA was extracted using the Quick DNA/RNA MagBead kit (Zymo Research, Irvine, CA; Supplementary Methods section in Supplementary Data).

### PDGFRα *in vitro* analyses

PDGFRα expression upregulation by prophylactic prednisolone was assessed in human and mouse primary hepatocytes *in vitro* (Supplementary Methods section in Supplementary Data). The effect of PDGFRα on AAV transduction was confirmed by knockdown in HepG2 cells with a short hairpin RNA (shRNA; Supplementary Methods section in Supplementary Data).

### Statistical methods

Serum hA1AT was analyzed using a Student's *t*-test at each time point after log_10_ transformation. Benjamini–Hochberg-adjusted *p* values were calculated for differential expression and gene set enrichment analyses in RNAseq experiments and Olink proteomic analysis using *p* < 0.05. Plasma FVIII protein and liver hFVIII-SQ vector genome copies were analyzed by a Student's *t*-test. Plasma PDGFα/α, complement system activation, and immunohistochemistry staining intensity were analyzed using one-way analysis of variance (ANOVA) and a Tukey's multiple comparison test. PDGFRα and PDGFα normalized expression were compared using a Welch's unpaired *t*-test.

## RESULTS

### Prophylactic prednisolone treatment increases transgene expression and vector DNA

In the 12-week study, mice who received prophylactic prednisolone on either day −1 or 0 had significantly higher serum hA1AT protein than nonprednisolone-treated mice from week 6 onward (Supplementary Fig. S1A). Serum protein was ∼1.5- to 2-fold higher for mice receiving prednisolone at either time point in weeks 6 through 12 than for nontreated mice; prophylactic prednisolone appeared to lower interindividual variability by reducing the number of mice with low expression (Fig. 1B).

More hepatocytes stained positive for vector DNA in mice that received prophylactic prednisolone versus water on either day −1 or 0 at weeks 4 and 12 (Supplementary Fig. S1B), although significance was not reached. A clear trend for more overall and full-length vector genomes was also detected in prednisolone-treated mice compared with nonprednisolone-treated mice (Fig. 1C and Supplementary Fig. S1C). Similarly, after dosing with AAV5-hFVIII-SQ, significantly more full-length vector DNA (*p* < 0.05) was present in the livers of NHPs that received prophylactic methylprednisolone and rituximab than in those that did not (Supplementary Fig. S2).

In mice, serum hA1AT protein levels correlated significantly (*r* = 0.68, *p* < 0.0001) with full-length vector genomes in the liver at week 12 (Fig. 1D). Transgene RNA levels also correlated with vector DNA (*r* = 0.49, *p* < 0.01) and transgene protein (*r* = 0.59, *p* < 0.001) at 12 weeks (Supplementary Fig. S1D, E). Overall, prophylactic corticosteroids improved AAV5-mediated transgene expression by increasing levels of functional full-length vector DNA in hepatocytes, giving rise to more RNA and protein.

### Prophylactic prednisolone promotes transduction within 24 h of AAV dosing

Previously, initiation of prednisolone 1 week after AAV5-hFVIII-SQ dosing did not modulate transgene expression,^22^ while here, initiating prednisolone before AAV dosing resulted in improved transgene expression. Hence, we hypothesized that the mechanism promoting AAV-mediated expression in response to prophylactic prednisolone occurs early and investigated potential mechanisms active immediately after AAV and steroid dosing.^22^ Mice received a single treatment of prednisolone or water 2 h before dosing with AAV or vehicle (Fig. 2A), and blood and livers were collected at 2 and 24 h.

First, we identified pathways impacted by AAV transduction or prednisolone treatment using RNAseq. Differential expression analyses were structured as treatment versus control, and differentially expressed genes (adjusted *p* < 0.05 and log_2_-fold change >1.5) identified (Supplementary Table S1). In mice treated with AAV5-HLP-hA1AT versus control, 115 and 231 genes were upregulated and downregulated, respectively, at 2 h postdose; by 24 h postdose, 36 and 160 genes were upregulated and downregulated, respectively. Significantly upregulated pathways, as indicated by NES, included mTORC1 signaling, complement pathway, interleukin (IL)-6, JAK-STAT3 signaling, and DNA repair pathways (Table 1).

**Table 1.** Enrichment of hallmark gene sets for the effect of adeno-associated virus at 2 and 24 hours postdose

Enrichment of Hallmark Gene Sets	2 H		24 H		References
	NES	Adjusted* p*-Value	NES	Adjusted* p*-Value	
Upregulation					
mTORC1 signaling	2	2.80E–07	1.79	0.0000646	
Protein secretion	2	2.18E–05	2	0.000087	
E2F targets	1.4	0.0522	1.78	9.15E–05	
Fatty acid metabolism	1.82	0.000144	—	—	
Complement	1.56	0.00639	—	—	PMID: 33393506
Peroxisome	1.55	0.0286	—	—	
Hypoxia	1.42	0.0522	—	—	
Myc targets v1			2.41	1.29E–12	
IL-6 JAK-STAT3 signaling	—	—	1.61	0.0271	
Unfolded protein response	—	—	1.59	0.0227	
Myc targets v2	—	—	1.59	0.0539	
UV response up	−2.53	1.22E–15	1.52	0.0204	
Estrogen response late	−2.18	1.87E–12	1.4	0.0327	
DNA repair	—	—	1.41	0.0398	
Downregulation					
Myogenesis	—	—	−1.46	0.0221	
UV response down	—	—	−1.41	0.0539	

NES determines whether a gene set is moving up (positively regulated) or down (negatively regulated) the gene rankings when comparing mice treated with prednisolone+AAV5-HLP-hA1AT versus mice treated with prednisolone+vehicle (*n* = 10/group). These curated data were sorted by NES.

AAV, adeno-associated virus; AAV5-HLP-hA1AT, AAV serotype 5 hybrid liver promoter human α1-antitrypsin; DNA, deoxyribonucleic acid; E, exponential; E2F, E2 factor; IL-6, interleukin-6; JAK STAT3, JAK STAT pathway 3; JAK, Janus kinase; mTORC1, mammalian target of rapamycin complex 1; NES, normalized enrichment score; PMID, PubMed ID; STAT, signal transducer and activator of transcription; UV, ultraviolet; v, vector.

### Prednisolone treatment suppresses innate immune responses

For mice treated with prophylactic prednisolone versus control before AAV dosing, 863 and 1561 genes were significantly upregulated and downregulated, respectively, at 2 h postdose. By 24 h, only 87 and 173 genes were upregulated and downregulated, respectively. Inflammatory response pathways, such as interferon-α and tumor necrosis factor-α/nuclear factor-κB signaling, were downregulated by prednisolone as indicated by NES (Table 2). We specifically examined IL-1β expression as a marker of the inflammasome and found it was significantly downregulated by prednisolone (Supplementary Fig. S3A).

**Table 2.** Enrichment of hallmark gene sets for the effect of prophylactic prednisolone at 2 and 24 hours postdosing with adeno-associated virus

Enrichment of Hallmark Gene Sets	2 H		24 H		References
	NES	Adjusted* p*-Value	NES	Adjusted* p*-Value	
Upregulation					
Adipogenesis	1.73	9.51E–05	1.47	0.0102	PMID: 31890737
Fatty acid metabolism	1.63	0.00265	1.59	0.00596	PMID: 1249197, 18434349
Protein secretion	1.65	0.00617	1.88	0.000265	
Myc targets v1	2.4	1.21E–15	—	—	PMID: 9886401
Myc targets v2	2.28	1.51E–07	—	—	PMID: 9886401
Oxidative phosphorylation	2.17	2.46E–10	—	—	PMID: 11129090, 15075441
Peroxisome	1.58	0.0125	—	—	PMID: 9142695 (review)
Unfolded protein response	1.74	0.00175	—	—	PMID: 23650437
Heme metabolism	—	—	1.79	8.74E–05	PMID: 5231752
Bile acid metabolism	—	—	1.71	0.0021	PMID: 24681341
Hypoxia	—	—	1.47	0.0255	PMID: 31524075
Downregulation					
IFN-α response	−2.53	1.22E–15	−2.14	2.3E–06	PMID: 11544466, 20679482
IFN-γ response	−2.18	1.87E–12	−2.16	1.05E–09	PMID: 12707366
Allograft rejection	—	—	−2.08	5.62E–08	PMID: 781384, 29427591
Cholesterol homeostasis	—	—	−1.72	0.0024	PMID: 24681341
Inflammatory response	—	—	−1.68	0.000493	PMID: 29479352
Myc targets v2	—	—	−1.66	0.0102	
TNF-α signaling via NF-κB	—	—	−1.6	0.00294	PMID: 15143169
KRAS signaling up	—	—	−1.59	0.0021	
IL-6 JAK STAT3 signaling	—	—	−1.56	0.0165	PMID: 21455087
Apoptosis	—	—	−1.54	0.00592	PMID: 24709697
Reactive oxygen species pathway	—	—	−1.5	0.046	PMID: 12117619

NES determines whether a gene set is moving up (positively regulated) or down (negatively regulated) the gene rankings when comparing mice treated with prednisolone+AAV5-HLP-hA1AT versus mice treated with water+AAV5-HLP-hA1AT (*n* = 10/group). These curated data were sorted by NES.

IFN-α, interferon-alpha; IFN-γ, interferon-gamma; KRAS, Kirsten rat sarcoma virus; NF-κB, nuclear factor kappa-light-chain-enhancer of activated B cells; TNF-α, tumor necrosis factor-alpha.

We used an exploratory Olink mouse panel (Uppsala, Sweden) to measure the effect of prophylactic prednisolone on plasma proteins at 2 and 24 h post-AAV dose. Normalized protein expression of most inflammatory response proteins was significantly suppressed by prophylactic prednisolone at both time points (*p* < 0.05; Supplementary Tables S2 and S3), but cytokines were upregulated by AAV treatment (*p* < 0.05; Supplementary Tables S4 and S5). However, serum ALT levels indicating liver damage were not significantly elevated with or without prednisolone (Supplementary Fig. S4A, B).

In addition, despite RNAseq data showing complement activation by AAV in the liver (Supplementary Table S6), there were no significant differences in plasma C3b levels between treatments (Supplementary Fig. S3B).

### Prednisolone treatment upregulates the AAV5 coreceptor PDGFRα

Because prophylactic prednisolone increased liver vector DNA, we hypothesized it enhances initial transduction efficacy. We thus investigated mechanisms involved in the initial uptake of AAV vectors. AAV5 vectors interact with extracellular *N*-glycans before binding to the essential multiserotype AAV receptor (AAVR) and the AAV5 coreceptors PDGFRα and PDGFRβ on the cell surface.^30–38^

Liver levels of *N*-glycans in mice at 2 and 24 h were not affected by prophylactic prednisolone (Supplementary Fig. S5). In our RNAseq data set, PDGFRα expression at 2 h post-AAV dose was significantly higher in livers of mice that received prednisolone versus water before AAV treatment, and PDGFα, a ligand for PDGFR, was significantly lower in mice that received prednisolone versus water before AAV dosing (Fig. 2B). However, differences did not persist to 24 h. AAVR or PDGFRβ expression did not significantly differ (data not shown).

Secreted PDGFα forms homodimer (PDGFα/α) or PDGFβ heterodimer complexes (PDGFα/β), and so, to confirm RNAseq data, circulating ligands were assessed using an ELISA for PDGFα/α and an Olink assay for PDGFβ/β (both also detect PDGFα/β). At 2 h post-AAV, plasma levels of PDGF ligands were lower, although not significant, in mice that received prophylactic prednisolone versus vehicle (Fig. 2C). Mass spectrometry of total liver homogenate samples found no significant difference between groups in PDGFRα (data not shown). As liver homogenate may include lysates from multiple cell types that could dilute signals, we also used immunohistochemistry to detect PDGFRα expression on hepatocytes. Analysis confirmed a significantly higher PDGFRα protein in a subset of hepatocytes at 2 h post-AAV dosing in mice treated with prophylactic prednisolone versus vehicle (Fig. 2D).

### Prophylactic prednisolone increases AAV5 uptake

**Figure 3.**
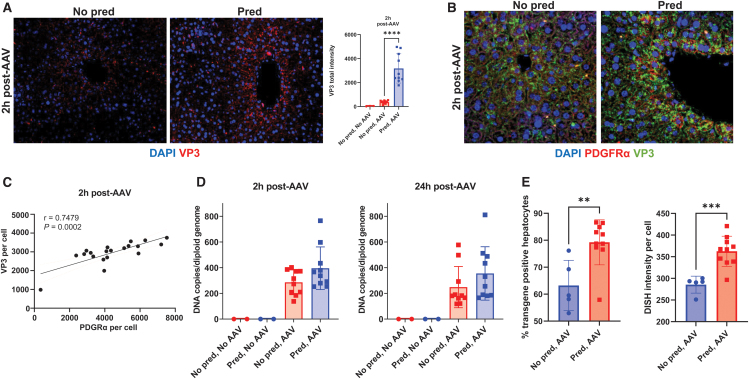
Upregulation of PDGFRα expression in hepatocytes correlates with increased AAV5 transduction. **(A)** AAV5 uptake into hepatocytes 2 h following AAV5-HLP-hA1AT administration with and without prophylactic prednisolone treatment as indicated with VP3. **(B)** PDGFRα and AAV5 uptake (via VP3) coincide in the same hepatocytes 2 h following AAV5-HLP-hA1AT administration with and without prophylactic prednisolone treatment. **(C)** Pearson correlation of PDGFRα and VP3 intensity in hepatocytes 2 h following AAV5-HLP-hA1AT administration with and without prophylactic prednisolone treatment. **(D)** ddPCR analysis of vector genome levels in liver homogenates 2 and 24 h following AAV5-HLP-hA1AT administration with and without prophylactic prednisolone treatment. **(E)** *In situ* hybridization analysis of vector genome levels in hepatocytes 1 week following AAV5-HLP-hA1AT administration with and without prophylactic prednisolone treatment. ****p < 0.0001; ***p < 0.001; **p < 0.05. Bar graphs show mean ± SD; Symbols are individual data points. Statistical analysis was performed using one-way ANOVA followed by a Tukey's multiple comparison test **(A)** or unpaired *t*-test **(D, E)**. In **(A, B)**, images are at 20 × magnification. AAV denotes AAV5-HLP-hA1AT dosing. DISH, DNA in situ hybridization; VP3, viral protein 3.

At 2 h postdose, using immunohistochemistry, intracellular VP3—an AAV5 viral capsid protein—was significantly higher with prednisolone treatment (Fig. 3A), consistent with increased uptake of AAV by cells.^39,40^ Furthermore, microscopy of VP3 and PDGFRα together showed that both were found in the same hepatocytes following AAV5 administration (Fig. 3B). PDGFRα staining intensity was significantly correlated (*r* = 0.7479, *p* < 0.0005) with VP3 intensity within hepatocytes at 2 h postdose (Fig. 3C).

In addition, prophylactic prednisolone in mice increased transgene DNA per cell as measured by ddPCR in the liver homogenate (Fig. 3D); however, these differences did not reach statistical significance. At the 2- and 24-h time points, the ISH signals for vector DNA were saturating, rendering quantitative analyses unfeasible. Therefore, one additional cohort was generated and taken down 1 week post-AAV dosing for ISH. The mean percentage of hepatocytes stained positive for vector DNA was significantly higher in mice treated with prophylactic prednisolone (79.2%) versus control (63.2%; Fig. 3E). Increased liver vector genomes resulted from both a higher percentage of hepatocytes taking up vector DNA and more uptake per hepatocyte (Fig. 3E). These results suggest that acute upregulation of PDGRFα proximal to AAV dosing increases levels of vector DNA within hepatocytes.

### PDGFRα expression variation may contribute to intersubject variability

**Figure 4.**
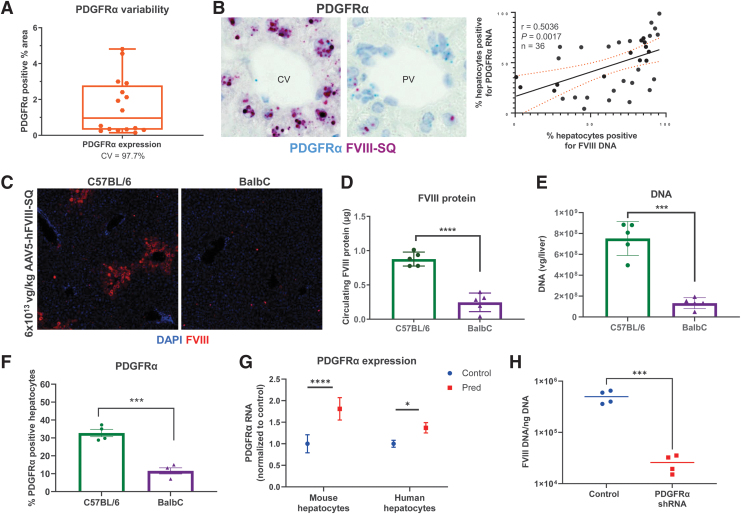
Relationship between PDGFRα and AAV5 transduction. **(A)** Variation in endogenous PDGFRα levels in naive C57BL/6 mice livers using fluorescence imaging technique and PDGFRα variability. **(B)** AAV5-FVIII-SQ vector genomes correlate with PDGFRα expression hepatocytes of C57BL/6 mice treated with AAV5-hFVIII-SQ vector. Results in C57BL/6 and Balb/C mice treated with AAV5-hFVIII-SQ vector. **(C)** Hepatocytes expressing the transgene protein hFVIII-SQ, **(D)** circulating hFVIII-SQ protein, **(E)** liver vector DNA levels, and **(F)** percentage of hepatocytes expressing PDGFRα receptor. **(G)** Prednisolone treatment upregulated PDGFRα in human and murine hepatocytes. **(H)** PDGFRα knockdown decreased levels of AAV5-FVIII-SQ vector genomes in HepG2 cells. *p < 0.05, ***p < 0.001, ****p < 0.0001. Bar graphs show mean ± SD; symbols are individual data points. For **(B)**, C57BL/6 mice were administered 6 × 10^13^ vg/kg AAV5-hFVIII-SQ. For **(C–F)**, C57BL/6 and Balb/C mice were administered 6 × 10^13^ vg/kg AAV5-hFVIII-SQ. Liver transgene DNA, circulating human FVIII protein, and PDGFRα were evaluated at 5 weeks postdose. **(B)** Shows Pearson correlation. Statistical significance for **(D–F)** was calculated using an unpaired *t*-test. AAV5, AAV serotype 5; CV, coefficient of variation; FVIII, factor VIII; FVIII-SQ, B-domain–deleted FVIII; hFVIII, human FVIII; shRNA, short hairpin ribonucleic acid.

We assessed potential contributions of individual natural variation in PDGFRα expression to variable transgene expression by comparing outcomes after AAV5-hFVIII-SQ dosing in C5BL/6 and BalbC mice. In C57BL/6 mice naive to AAV or corticosteroid treatment, endogenous PDGFRα liver expression is highly variable (Fig. 4A); after AAV5-hFVIII-SQ dosing, the percentage of hepatocytes stained positive for PDGFRα significantly correlated with those positive for vector genome DNA (Fig. 4B).

When comparing outcomes among laboratory mouse strains after AAV5-hFVIII-SQ treatment, BalbC mice had fewer hepatocytes expressing transgene protein (Fig. 4C), lower circulating transgene protein product (Fig. 4D), and lower levels of vector DNA (Fig. 4E) than C57BL/6 mice. Interestingly, a significantly lower percentage of hepatocytes expressed PDGFRα (but not PDGFRβ; data not shown) in BalbC mice versus C57BL/6 mice (Fig. 4F).

### *In vitro* results confirm prednisolone increases PDGFRα expression, affecting AAV5 transduction

To assess if prednisolone affects PDGFRα expression in human hepatocytes, we treated primary hepatocytes with prednisolone. PDGRFα transcripts significantly increased in both human and murine hepatocytes (Fig. 4G), as well as monkey and dog hepatocytes (data not shown). Furthermore, shRNA knockdown of PDGFRα in the human hepatocyte HepG2 cell line significantly decreased AAV5 transduction (Fig. 4H). Altogether, prophylactic prednisolone increases PDGFRα expression, resulting in increased transduction of AAV5 vectors, leading to more vector DNA in hepatocytes, and improved transgene expression.

## DISCUSSION

We investigated whether prophylactic prednisolone before AAV administration affected transgene expression. In mice, prophylactic prednisolone before AAV vector dosing increased steady-state transgene serum protein levels over 12 weeks and appeared to reduce interindividual variability in transgene protein expression. In mice and NHPs, prophylactic corticosteroids increased vector genome copy numbers in the liver. We then investigated mechanisms of improved transgene expression focusing on time points proximal to AAV dosing, since AAV uptake and acute innate immune responses occur rapidly following AAV administration.^41^ Overall, prophylactic prednisolone increased the uptake of AAV5 vectors by suppressing innate immune responses and upregulating the expression of the AAV5 receptor PDGFRα. Together, these mechanisms increased vector genomes available to assemble into stable, full-length structures mediating long-term transgene expression.

Our RNAseq analyses reiterate that AAV transduction activates innate immune responses and prednisolone treatment modulates those responses through known pathways.^22,42–61^ By decreasing acute anti-AAV innate immunity, prophylactic steroids may reduce vector genome degradation and thus preserve more genomes postdosing, increasing available genomes to form full-length genome structures for transgene expression.

Surprisingly, prophylactic corticosteroids also modulated cellular mechanisms involved in receptor-mediated endocytosis of AAV5; this may explain why corticosteroids administered 1 week post-AAV have little effect.^22^ Consistent with the role of PDGFRα in AAV5 transduction,^30,36,38^ PDGFRα levels correlated with AAV5 transduction in laboratory mice strains, although differential immune responses may also have contributed.^62–64^ Knockdown of PDGFRα expression resulted in lower vector DNA levels in transduced hepatocytes *in vitro*. This suggests that prophylactic prednisolone increases the expression of the AAV5 coreceptor PDGFRα and decreases the expression of the competitive PDGF ligand complexes, resulting in an overall higher AAV5 uptake by hepatocytes. A significantly higher PDGFRα protein expression was present in hepatocytes 2 h after AAV dosing in mice treated with prophylactic prednisolone, coinciding with rapid cellular uptake of AAV vectors postdosing.^39,40^

Collectively, these data indicate that variation in AAV5 receptors, particularly PDGFRα, may be a host factor contributing to the variable response seen in gene therapy trials. Although PDGFRα expression is variable in human livers (Supplementary Fig. S6), its contribution to response differences in clinical trials is unknown.^65^ Insights will be provided by the ongoing phase 3 trial evaluating valoctocogene roxaparvovec with prophylactic corticosteroids in participants with hemophilia A (NCT04323098).

A limitation of this research is the use of a mouse model for human disease, given immune system differences.^66^ We also did not consider the effect of vector dose, adaptive immunity, or mechanisms after capsid uptake.

Overall, prophylactic corticosteroids before dosing with AAV5-based gene therapy increase transgene expression in mice through multiple complementary mechanisms, including innate immune suppression and increased AAV5 uptake by hepatocytes. Therefore, prophylactic corticosteroids may represent an actionable strategy for increasing transgene expression and reducing interindividual variability in response to AAV5-mediated gene therapy.

## Supplementary Material

Supplemental data

## DATA AND MATERIALS AVAILABILITY

Materials and protocols will be distributed to qualified scientific researchers for noncommercial, academic purposes. The AAV5-hFVIII-SQ vector and sequence are part of an ongoing development program, and they will not be shared.

## References

[1] Nathwani AC, Tuddenham EG, Rangarajan S, et al. Adenovirus-associated virus vector–mediated gene transfer in hemophilia B. N Engl J Med 2011;365:2357–2365. 22149959 10.1056/NEJMoa1108046 PMC3265081

[2] George LA, Monahan PE, Eyster ME, et al. Multiyear factor VIII expression after AAV gene transfer for hemophilia A. N Engl J Med 2021;385:1961–1973. 34788507 10.1056/NEJMoa2104205 PMC8672712

[3] Ronzitti G, Gross D-A, Mingozzi F. Human immune responses to adeno-associated virus (AAV) vectors. Front Immunol 2020;11:670. 32362898 10.3389/fimmu.2020.00670 PMC7181373

[4] George LA, Ragni MV, Rasko JE, et al. Long-term follow-up of the first in human intravascular delivery of AAV for gene transfer: AAV2-hFIX16 for severe hemophilia B. Mol Ther 2020;28:2073–2082. 32559433 10.1016/j.ymthe.2020.06.001 PMC7474338

[5] Muhuri M, Maeda Y, Ma H, et al. Overcoming innate immune barriers that impede AAV gene therapy vectors. J Clin Invest 2021;131:e143780. 33393506 10.1172/JCI143780 PMC7773343

[6] Rabinowitz J, Chan YK, Samulski RJ. Adeno-associated virus (AAV) versus immune response. Viruses 2019;11:102. 30691064 10.3390/v11020102 PMC6409805

[7] Mingozzi F, High KA. Immune responses to AAV vectors: Overcoming barriers to successful gene therapy. Blood 2013;122:23–36. 23596044 10.1182/blood-2013-01-306647 PMC3701904

[8] Rangarajan S, Walsh L, Lester W, et al. AAV5-Factor VIII gene transfer in severe hemophilia A. N Engl J Med 2017;377:2519–2530. 29224506 10.1056/NEJMoa1708483

[9] Ozelo MC, Mahlangu J, Pasi KJ, et al. Valoctocogene roxaparvovec gene therapy for hemophilia A. N Engl J Med 2022;386:1013–1025. 35294811 10.1056/NEJMoa2113708

[10] Mendell JR, Al-Zaidy S, Shell R, et al. Single-dose gene-replacement therapy for spinal muscular atrophy. N Engl J Med 2017;377:1713–1722. 29091557 10.1056/NEJMoa1706198

[11] Samulski RJ, Muzyczka N. AAV-mediated gene therapy for research and therapeutic purposes. Annu Rev Virol 2014;1:427–451. 26958729 10.1146/annurev-virology-031413-085355

[12] Walport MJ. Complement. First of two parts. N Engl J Med 2001;344:1058–1066. 11287977 10.1056/NEJM200104053441406

[13] Hui DJ, Edmonson SC, Podsakoff GM, et al. AAV capsid CD8+ T-cell epitopes are highly conserved across AAV serotypes. Mol Ther Methods Clin Dev 2015;2:15029. 26445723 10.1038/mtm.2015.29 PMC4588448

[14] Vandamme C, Adjali O, Mingozzi F. Unraveling the complex story of immune responses to AAV vectors trial after trial. Hum Gene Ther 2017;28:1061–1074. 28835127 10.1089/hum.2017.150 PMC5649404

[15] Williams DM. Clinical pharmacology of corticosteroids. Respir Care 2018;63:655–670. 29794202 10.4187/respcare.06314

[16] Coutinho AE, Chapman KE. The anti-inflammatory and immunosuppressive effects of glucocorticoids, recent developments and mechanistic insights. Mol Cell Endocrinol 2011;335:2–13. 20398732 10.1016/j.mce.2010.04.005 PMC3047790

[17] Pasi KJ, Rangarajan S, Mitchell N, et al. Multiyear follow-up of AAV5-hFVIII-SQ gene therapy for hemophilia A. N Engl J Med 2020;382:29–40. 31893514 10.1056/NEJMoa1908490

[18] Pasi KJ, Laffan M, Rangarajan S, et al. Persistence of haemostatic response following gene therapy with valoctocogene roxaparvovec in severe haemophilia A. Haemophilia 2021;27:947–956. 34378280 10.1111/hae.14391 PMC9291073

[19] Mahlangu J, Kaczmarek R, von Drygalski A, et al. Two-year outcomes of valoctocogene roxaparvovec therapy for hemophilia A. N Engl J Med 2023;388:694–705. 36812433 10.1056/NEJMoa2211075

[20] Chai Z, Zhang X, Dobbins AL, et al. Optimization of dexamethasone administration for maintaining global transduction efficacy of adeno-associated virus serotype 9. Hum Gene Ther 2019;30:829–840. 30700148 10.1089/hum.2018.233 PMC6648223

[21] Cramer ML, Shao G, Rodino-Klapac LR, et al. Induction of T-cell infiltration and programmed death ligand 2 expression by adeno-associated virus in rhesus macaque skeletal muscle and modulation by prednisone. Hum Gene Ther 2017;28:493–509. 28345428 10.1089/hum.2016.113 PMC5488353

[22] Zhang L, Handyside B, Murphy R, et al. Prednisolone does not regulate factor VIII expression in mice receiving AAV5-hFVIII-SQ: Valoctocogene roxaparvovec. Mol Ther Methods Clin Dev 2020;17:13–20. 31890737 10.1016/j.omtm.2019.11.007 PMC6923509

[23] Sihn CR, Handyside B, Liu S, et al. Molecular analysis of AAV5-hFVIII-SQ vector-genome-processing kinetics in transduced mouse and nonhuman primate livers. Mol Ther Methods Clin Dev 2022;24:142–153. 35036471 10.1016/j.omtm.2021.12.004 PMC8749450

[24] Nathwani AC, Reiss UM, Tuddenham EG, et al. Long-term safety and efficacy of factor IX gene therapy in hemophilia B. N Engl J Med 2014;371:1994–2004. 25409372 10.1056/NEJMoa1407309 PMC4278802

[25] Chiuchiolo MJ, Kaminsky SM, Sondhi D, et al. Intrapleural administration of an AAVrh.10 vector coding for human alpha1-antitrypsin for the treatment of alpha1-antitrypsin deficiency. Hum Gene Ther Clin Dev 2013;24:161–173. 24191907 10.1089/humc.2013.168

[26] De B, Heguy A, Leopold PL, et al. Intrapleural administration of a serotype 5 adeno-associated virus coding for alpha1-antitrypsin mediates persistent, high lung and serum levels of alpha1-antitrypsin. Mol Ther 2004;10:1003–1010. 15564132 10.1016/j.ymthe.2004.08.022

[27] The Genome Reference Consortium. Mouse reference genome GRCm39; 2022. Available from: https://www.ncbi.nlm.nih.gov/grc/mouse [Last accessed: March 30, 2022].

[28] Liberzon A, Birger C, Thorvaldsdóttir H, et al. The molecular signatures database hallmark gene set collection. Cell Syst 2015;1:417–425. 26771021 10.1016/j.cels.2015.12.004 PMC4707969

[29] Subramanian A, Tamayo P, Mootha VK, et al. Gene set enrichment analysis: A knowledge-based approach for interpreting genome-wide expression profiles. Proc Natl Acad Sci U S A 2005;102:15545–15550. 16199517 10.1073/pnas.0506580102 PMC1239896

[30] Di Pasquale G, Davidson BL, Stein CS, et al. Identification of PDGFR as a receptor for AAV-5 transduction. Nat Med 2003;9:1306–1312. 14502277 10.1038/nm929

[31] Summerford C, Samulski RJ. AAVR: A multi-serotype receptor for AAV. Mol Ther 2016;24:663–666. 27081719 10.1038/mt.2016.49 PMC4886949

[32] Large EE, Silveria MA, Zane GM, et al. Adeno-associated virus (AAV) gene delivery: Dissecting molecular interactions upon cell entry. Viruses 2021;13:1336. 34372542 10.3390/v13071336 PMC8310307

[33] Afione S, DiMattia MA, Halder S, et al. Identification and mutagenesis of the adeno-associated virus 5 sialic acid binding region. J Virol 2015;89:1660–1672. 25410855 10.1128/JVI.02503-14 PMC4300766

[34] Seiler MP, Miller AD, Zabner J, et al. Adeno-associated virus types 5 and 6 use distinct receptors for cell entry. Hum Gene Ther 2006;17:10–19. 16409121 10.1089/hum.2006.17.10

[35] Mietzsch M, Broecker F, Reinhardt A, et al. Differential adeno-associated virus serotype-specific interaction patterns with synthetic heparins and other glycans. J Virol 2014;88:2991–3003. 24371066 10.1128/JVI.03371-13 PMC3958061

[36] Pilz IH, Di Pasquale G, Rzadzinska A, et al. Mutation in the platelet-derived growth factor receptor alpha inhibits adeno-associated virus type 5 transduction. Virology 2012;428:58–63. 22520943 10.1016/j.virol.2012.03.004 PMC3350617

[37] Pillay S, Zou W, Cheng F, et al. Adeno-associated virus (AAV) serotypes have distinctive interactions with domains of the cellular AAV receptor. J Virol 2017;91:e00391–00317. 28679762 10.1128/JVI.00391-17 PMC5571256

[38] Pillay S, Meyer N, Puschnik A, et al. An essential receptor for adeno-associated virus infection. Nature 2016;530:108–112. 26814968 10.1038/nature16465 PMC4962915

[39] Bartlett JS, Wilcher R, Samulski RJ. Infectious entry pathway of adeno-associated virus and adeno-associated virus vectors. J Virol 2000;74:2777–2785. 10684294 10.1128/jvi.74.6.2777-2785.2000 PMC111768

[40] Nonnenmacher M, Weber T. Intracellular transport of recombinant adeno-associated virus vectors. Gene Ther 2012;19:649–658. 22357511 10.1038/gt.2012.6 PMC4465241

[41] Shao W, Earley LF, Chai Z, et al. Double-stranded RNA innate immune response activation from long-term adeno-associated virus vector transduction. JCI Insight 2018;3:e120474. 29925692 10.1172/jci.insight.120474 PMC6124417

[42] Krotkiewski M, Blohmé B, Lindholm N, et al. The effects of adrenal corticosteroids on regional adipocyte size in man. Int J Clin Endocrinol Metab 1976;42:91–97. 10.1210/jcem-42-1-91 1249197

[43] Macfarlane DP, Forbes S, Walker BR. Glucocorticoids and fatty acid metabolism in humans: Fuelling fat redistribution in the metabolic syndrome. J Endocrinol 2008;197:189–204. 18434349 10.1677/JOE-08-0054

[44] Wang W, Wykrzykowska J, Johnson T, et al. A NF-κB/c-myc-dependent survival pathway is targeted by corticosteroids in immature thymocytes. J Immunol 1999;162:314–322. 9886401

[45] Morin C, Zini R, Simon N, et al. Low glucocorticoid concentrations decrease oxidative phosphorylation of isolated rat brain mitochondria: An additional effect of dexamethasone. Fundam Clin Pharmacol 2000;14:493–500. 11129090 10.1111/j.1472-8206.2000.tb00432.x

[46] Manczak M, Park BS, Jung Y, et al. Differential expression of oxidative phosphorylation genes in patients with Alzheimer's disease. Neuromolecular Med 2004;5:147–162. 15075441 10.1385/NMM:5:2:147

[47] Magalhães MM, Magalhães MC. Peroxisomes in adrenal steroidogenesis. Microsc Res Tech 1997;36:493–502. 9142695 10.1002/(SICI)1097-0029(19970315)36:6<493::AID-JEMT6>3.0.CO;2-J

[48] Das I, Png CW, Oancea I, et al. Glucocorticoids alleviate intestinal ER stress by enhancing protein folding and degradation of misfolded proteins. J Exp Med 2013;210:1201–1216. 23650437 10.1084/jem.20121268 PMC3674691

[49] Granick S, Kappas A. Steroid control of porphyrin and heme biosynthesis: A new biological function of steroid hormone metabolites. Proc Natl Acad Sci U S A 1967;57:1463. 5231752 10.1073/pnas.57.5.1463 PMC224495

[50] Out C, Dikkers A, Laskewitz A, et al. Prednisolone increases enterohepatic cycling of bile acids by induction of Asbt and promotes reverse cholesterol transport. J Hepatol 2014;61:351–357. 24681341 10.1016/j.jhep.2014.03.025

[51] Sandhu MS, Gray E, Kocherginsky M, et al. Prednisolone pretreatment enhances intermittent hypoxia-induced plasticity in persons with chronic incomplete spinal cord injury. Neurorehabil Neural Repair 2019;33:911–921. 31524075 10.1177/1545968319872992

[52] Shodell M, Siegal FP. Corticosteroids depress IFN-α–producing plasmacytoid dendritic cells in human blood. J Allergy Clin Immunol 2001;108:446–448. 11544466 10.1067/mai.2001.117928

[53] Flammer JR, Dobrovolna J, Kennedy MA, et al. The type I interferon signaling pathway is a target for glucocorticoid inhibition. Mol Cell Biol 2010;30:4564–4574. 20679482 10.1128/MCB.00146-10 PMC2950533

[54] Hu X, Li W-P, Meng C, et al. Inhibition of IFN-γ signaling by glucocorticoids. J Immunol 2003;170:4833–4839. 12707366 10.4049/jimmunol.170.9.4833

[55] Alarcon-Zurita A, Ladefoged J. Treatment of acute allograft rejection with high doses of corticosteroids. Kidney Int 1976;9:351–354. 781384 10.1038/ki.1976.41

[56] Christakoudi S, Runglall M, Mobillo P, et al. Steroid regulation: An overlooked aspect of tolerance and chronic rejection in kidney transplantation. Mol Cell Endocrinol 2018;473:205–216. 29427591 10.1016/j.mce.2018.01.021

[57] Negera E, Walker SL, Bobosha K, et al. The effects of prednisolone treatment on cytokine expression in patients with erythema nodosum leprosum reactions. Front Immunol 2018;9:189. 29479352 10.3389/fimmu.2018.00189 PMC5811481

[58] Hermoso MA, Matsuguchi T, Smoak K, et al. Glucocorticoids and tumor necrosis factor alpha cooperatively regulate toll-like receptor 2 gene expression. Mol Cell Biol 2004;24:4743–4756. 15143169 10.1128/MCB.24.11.4743-4756.2004 PMC416411

[59] Jules-Elysee KM, Lipnitsky JY, Patel N, et al. Use of low-dose steroids in decreasing cytokine release during bilateral total knee replacement. Reg Anesth Pain Med 2011;36:36–40. 21455087 10.1097/AAP.0b013e31820306c5

[60] Gruver-Yates AL, Cidlowski JA. Tissue-specific actions of glucocorticoids on apoptosis: A double-edged sword. Cells 2013;2:202–223. 24709697 10.3390/cells2020202 PMC3972684

[61] Sanner BM, Meder U, Zidek W, et al. Effects of glucocorticoids on generation of reactive oxygen species in platelets. Steroids 2002;67:715–719. 12117619 10.1016/s0039-128x(02)00024-7

[62] Watanabe H, Numata K, Ito T, et al. Innate immune response in Th1- and Th2-dominant mouse strains. Shock 2004;22:460–466. 15489639 10.1097/01.shk.0000142249.08135.e9

[63] Fornefett J, Krause J, Klose K, et al. Comparative analysis of humoral immune responses and pathologies of BALB/c and C57BL/6 wildtype mice experimentally infected with a highly virulent *Rodentibacter pneumotropicus* (*Pasteurella pneumotropica*) strain. BMC Microbiol 2018;18:45. 29848308 10.1186/s12866-018-1186-8 PMC5977748

[64] Bleul T, Zhuang X, Hildebrand A, et al. Different innate immune responses in BALB/c and C57BL/6 strains following corneal transplantation. J Innate Immun 2021;13:49–59. 32906119 10.1159/000509716 PMC7879253

[65] Broad Institute of MIT and Harvard. Bulk tissue gene expression for PDGFRA. GTEx Analysis Release V8 (dbGaP Accession phs000424.v8.p2); 2021. Available from: https://gtexportal.org/home/gene/PDGFRA [Last accessed: March 8, 2022].

[66] Zschaler J, Schlorke D, Arnhold J. Differences in innate immune response between man and mouse. Crit Rev Immunol 2014;34:433–454. 25404048

