# Supplementary material for: Prophylactic Prednisolone Promotes AAV5 Hepatocyte Transduction Through the Novel Mechanism of AAV5 Coreceptor Platelet-Derived Growth Factor Receptor Alpha Upregulation and Innate Immune Suppression
Source: Hum Gene Ther. 2024 Jan 16;35(1-2):36–47. doi: 10.1089/hum.2023.065 (PMC10818045; doi:10.1089/hum.2023.065)
Supplement: Supplemental data [file Suppl_Data.docx]

# Supplemental material

# Supplemental methods

## Non-human primates study design

This study has been previously described.^1^ Briefly, juvenile (3–4 years) cynomolgus monkeys negative for adeno-associated virus serotype 5 (AAV5) total antibodies and neutralizing antibodies received an intravenous bolus injection of 6x10^13^ vg/kg valoctocogene roxaparvovec (AAV5-hFVIII-SQ). Animals received either no immunosuppressants or intravenous rituximab and intramuscular methylprednisolone beginning 7 days prior to vector dosing. Animals who received no immunosuppressants were euthanized at week 13 (n = 4), and animals who received immunosuppressants were euthanized at week 26 (n = 4). Liver samples were taken for assessment of vector DNA.

## Proteomics analysis using Olink

The mice exploratory panel was simultaneously analyzed for 92 protein biomarkers using the Olink Target 96 Mouse Exploratory assay (Olink Proteomics Inc., Waltham, MA). Normalized protein expression (NPX; log_2_ scale) for treatment groups was analyzed using the Olink insights tool (<https://olinkproteomics.shinyapps.io/OlinkInsightsStatAnalysis/>). *P* values are from t-tests performed on NPX values and are adjusted for multiple testing using the Benjamini-Hochberg correction in Olink Insights Stat Analysis. The cutoff for inclusion was *P* value <0.05. Mouse ethylenediaminetetraacetic acid plasma samples from the short-term mouse study were analyzed with the Olink Target 96 Mouse Exploratory panel.

## Complement system activity assay

Complement system activation was evaluated using plasma C3b levels as measured with a Hycult C3b ELISA kit HK216 (Wayne, PA), per the manufacturer’s instructions.

## Assessment of circulating alanine aminotransferase

Terminal plasma levels of alanine aminotransferase (ALT) were determined using 2 μL of serum/well with the ALT (MAK052) activity assay kit (Sigma-Aldrich, St. Louis, MO), according to the manufacturer’s instructions for fluorometric measurements. Fluorometric detection was performed with a FlexStation 3 multimode microplate reader (Molecular Devices, Sunnyvale, CA).

## N-glycome analysis

### Tissue preparation and homogenization

Mice livers were homogenized in 5:1 (µL:mg liver) Tissue Protein Extraction Reagent (Thermo Scientific, Waltham, MA) with a Bead Ruptor 24 Elite (Omni, International, Kennesaw, GA). Bicinchoninic acid protein assay (Thermo Scientific) was then performed to determine the average protein concentration for each of the samples.

### Filter-aided N-glycan separation isolation of glycoproteins for N-glycan release

Protein equivalents (250 µg) of homogenate were mixed with lysis buffer (8% sodium dodecyl sulphate/0.4% sodium deoxycholate/100 mM tris2-carboxyethyl phosphine/200 mM ammonium bicarbonate pH 8) in a 1:1 sample/lysis buffer volume ratio. The mixture was then heated at 90°C for 10 minutes. Once heated, the samples were cooled and processed according to the filter-aided N-glycan separation method described previously.^2^ The released N-glycans were dried overnight in a vacuum concentrator.

### Permethylation of saccharides

Dried N-glycans were resuspended in 200 µL dimethyl sulfoxide and were left to mix with micro-stir bars for 15 minutes. Once reconstituted, approximately 20 mg of solid sodium hydroxide power and 150 µL of methyl iodide were added to the samples and were allowed to react for 30 to 60 minutes, until samples appeared milky white. The reaction was quenched with 1 mL of water. A liquid-liquid extraction was performed with the addition of 1 mL of dichloromethane. The organic layer was retained and washed 4 more times with 2 mL water. The samples were then dried under nitrogen and prepared for matrix-assisted laser desorption/ionization time-of-flight (MALDI-TOF) analysis.

### MALDI-TOF & MALDI-TOF-tandem mass spectrometry of tissue samples

MALDI matrix (20 mg/mL Super DHB in 50:50 acetonitrile:3 mM NaCl in water) was mixed in a 1:1 ratio with the permethylated N-glycans after reconstituting them in liquid chromatography–mass spectrometry–grade water. Approximately 1 µL of each glycan-matrix mix was first spotted on the target plate and allowed to dry under vacuum before recrystallization in 0.5 µL of ethanol. A SYNAPT G2-Si HDMS MS (Waters Corporation, Milford, MA) was used in resolution mode for the analysis of these glycans. Prior to ionization, the system was calibrated to 5000 m/z using sodium iodide clusters to improve detection of larger analytes. Samples were continuously ionized using a spiral pattern over a time period of 2.8 minutes with a step rate of 50 ms. A laser power of 450 (arbitrary units) was used along with a repetition rate of 1000 Hz to capture the largest range of structures present. Any collision-induced dissociation performed was done using collision energies ranging from 120 to 140 V.

### In silico analysis of putative glycan structures using GlycoWorkbench

Putative structures were evaluated for matching mass over charge (m/z) values using the GlycoWorkbench software.^3^ These m/z values were then cross-referenced to previous publications at the consortium of functional glycomics.^4^ Detectable structures were defined as those that possessed intensities above 3 times baseline numerically, presented with clear isotopic clusters, and were within a 1-amu mass difference to the putative structure. These structures were then compared for significance using an unpaired t-test across treatment groups by normalizing each glycan in a sample to the total ion intensity of all glycans detected within that sample.

## Treatment of primary hepatocytes with prednisolone

Primary human hepatocytes (Lonza, cat# HUCPG) and primary murine hepatocytes were cultured in HCM hepatocyte culture media and MM maintenance media (Lonza, MM250), respectively. Cells were treated with 2.5 µg/mL of prednisolone and incubated for 24 hours at 37°C with 5% CO_2_. Total RNA was extracted using the RNeasy Micro Kit (Qiagen). Concentration of extracted RNA was measured using a NanoDrop 8000 spectrophotometer (Thermo Fisher Scientific) and then diluted to 200 ng/μL. For each sample, 2 μg RNA was reverse-transcribed to generate first-strand cDNA using SuperScript VILO Master Mix (Life Technologies, Carlsbad, CA, USA). Primers and probe targeting platelet-derived growth factor receptor alpha (PDGFRα; forward primer, AAGCTGAAGGACTGGG; reverse primer, GTCCTCTCTCTTGATGAAGG; FAM probe, ACCTGGGCAAGAGGAACAG) were used in the reaction. The reaction mixtures were prepared with 1x droplet digital polymerase chain reaction (ddPCR) Supermix for Probes (no dUTP; Bio‑Rad, Hercules, CA), primer sets (250 nM each), probes (900 nM each), and 5 μL of sample input in a final volume of 25 μL. Droplets were generated with the reaction mix and QX200 Droplet Generation Oil for Probes (Bio-Rad) using an Auto Droplet Generator (Bio-Rad). After droplet generation, 96-well PCR plates containing reaction mixtures were transferred to a C1000 Touch Thermal Cycler (Bio-Rad) for PCR reaction with the following cycling conditions: a cycle of 95°C for 10 minutes, 40 cycles of 95°C for 30 seconds and 58°C for 1 minute, followed by a cycle of 98°C for 10 minutes and 4°C hold. The samples were read using a QX200 droplet reader. Total concentration of targeted sequences, copies per μL, was processed using QuantaSoft software (Bio-Rad). All vector genome copies are normalized to total DNA input levels to obtain copies per ng DNA.

## PDGFRα knockdown in HepG2 cells

On day 0, 50,000 HepG2 cells (ATCC, Manassas, VA) were seeded in each well of a 24-well tissue culture plate (Corning, Corning, NY). Cells were seeded in complete Dulbecco’s Modified Eagle Medium (CDMEM; DMEM + 10% fetal bovine serum; Life Technologies, Carlsbad, CA). The next day, day 1, lentivirus at a multiplicity of infection (MOI) 5 was added in fresh CDMEM + polybrene (8 μg/mL; Millipore Sigma, Rockville, MD). A lentivirus driving expression of short hairpin RNA (shRNA) targeting PDGFRα (Millipore Sigma; Clone ID, TRCN0000001425) was used to knock down PDGFRα expression, and an empty vector lentivirus was used as control (Millipore Sigma). Lentivirus was left on the cells for 24 hours and then replaced with fresh CDMEM. On day 3, cells from 2 wells of the 24-well plate were counted using a Vi-CELL XR Cell Viability Analyzer (Beckman Coulter, San Diego, CA) and were used to determine the viral particles (AAV5-FVIII-SQ) necessary to achieve an MOI of 1x10^6^. CDMEM from the previous day was removed and cells were washed with Dulbecco's phosphate-buffered saline (DPBS) before addition of AAV in Hepatocyte Basal Medium (HBM; Lonza, Switzerland). Two wells that were treated with control lentivirus and the PDGFRα shRNA lentivirus were harvested to assess knockdown at this time point. On day 4, an equal volume of Hepatocyte Culture Medium (HBM + supplements) containing 200 µM etoposide (Enzo Life Sciences, Farmingdale, NY) was added to HBM + AAV from day 3. On day 5, media was removed, cells were washed with DPBS, and CDMEM was added. Two days later, day 7, all media was removed, and wells were washed with DPBS before being frozen at –80^o^C for subsequent RNA/DNA purification and ddPCR analysis. Total DNA was extracted from each well using an AllPrep DNA/RNA micro kit (Qiagen, Valencia CA). RNA and DNA were quantified using a NanoDrop 8000 spectrophotometer (Thermo Fisher Scientific, San Jose, CA). DNA was diluted to 15 ng/μL for downstream ddPCR analysis of FVIII-SQ copies per ng total DNA as previously described.^1^ For DNA analysis of FVIII-SQ, 0.075 ng DNA was used as input for ddPCR analysis. Four wells each treated with control lentivirus and PDGFRα shRNA lentivirus were assessed, and technical duplicates were averaged for each sample.

## Human α1-antitrypsin protein assay

Black MaxiSorp 96-well immuno plates (Thermo Fisher Scientific, Waltham, MA) were coated overnight at room temperature with the capture antibody at 400 ng/mL in DPBS. After washing with PBS (Corning, Corning NY) and 0.05% Tween-20 (Sigma-Aldrich, St. Louis, MO), all wells were blocked with 300 µL of reagent diluent (5% Tween-20 in PBS) for 1 hour at room temperature. After the blocking step, the plates were washed, and the diluted plasma samples were added along with standards and incubated at room temperature for 2 hours. Following this step, plates were washed, and the detection antibody was added at 150 ng/mL and incubated for 2 hours at room temperature. After another wash, the streptavidin-horseradish peroxidase (HRP) antibody working solution was added to all wells, and the plates were incubated for 20 minutes at room temperature. After a final wash, 100 μL of QuantaBlu fluorogenic peroxidase substrate (Thermo Fisher Scientific) was added per well, and the plates were incubated for 20 minutes on a shaker at room temperature protected from light. Subsequently, 100 μL of the QuantaBlu stop solution was added per well, and fluorescence was measured on a FlexStation 3 multimode plate reader (Molecular Devices, San Jose, CA) with excitation at 325 nm, emission at 420 nm, and a cutoff at 425 nm. Data were analyzed using SoftMaxPro 7.1 (Molecular Devices), exported to Excel spreadsheets (Excel 365, Microsoft Corporation) and further analyzed and plotted using Prism 8.4.3 (GraphPad, San Diego, CA).

*Droplet digital polymerase chain reaction procedures*

Quantities of vector genome forms in samples were measured with ddPCR, which captures individual DNA molecules in about twenty thousand water-oil emulsion droplets prior to PCR amplification with fluorescent tags. Individual droplets are then counted as negative or positive using fluorescence, and Poisson statistics are applied to the fraction of positive droplets to estimate the copy number of target DNA molecules per sample. In this analysis, drop-phase ddPCR was performed to detect and quantify the levels of paired target sequences together on a single DNA molecule to measure the contiguity of the DNA molecule, using two different fluorescent tags (FAM and HEX). The number of double-positive droplets is then calculated and total copy number of molecules with both target sequences are estimated using the software QuantaSoft™ (Bio-Rad, Hercules, CA), which provides as linkage. Full-length vector genomes were determined by the number of linkage copy of R1 and R11 amplicons, which overlap with the D segments of the ITR on the 5’ and 3’ ends of the genome, respectively.^25^ The copy number of endogenous gene *TFRC (*Transferrin receptor protein 1) was used a normalization reference for calculating vector copy numbers per diploid genome.

## Quantitative real-time PCR of hFVIII-SQ vector genomes

Levels of FVIII vector genome in liver genomic DNA were measured with a quantitative real-time PCR (qPCR) assay using a TaqMan DNA probe and primers specific to the human FVIII-SQ transgene. Approximately 20-µL reactions were set up with 2x iQ Multiplex Powermix (Bio-Rad Laboratories, Hercules, CA), 300 nM (final concentration) of forward and reverse primers (FVIII-SQ forward primer: ATGCACAGCATCAATGGCTA, FVIII-SQ reverse primer: CCATCTTGTGCTTGAAGGTG, Eurofins MWG Operon [Luxembourg]) and 150 nM (final concentration) of fluorescent probes (sequence: FAM-CCTGAGCATTGGGGCCCAGA-BHQ1, Eurofins MWG Operon) targeting FVIII-SQ transgene and approximately 100 ng of DNA. qPCR reactions were run on a Roche Light Cycler 480 II (Basel, Switzerland). Following denaturation at 95°C for 2 minutes, 50 thermal cycles were performed at 95°C for 3 seconds and 60°C for 20 seconds. Filters detecting fluorescence at 483 to 533 nm were used to detect amplicons corresponding to FVIII-SQ transgene. Cycle cross point (Cp) values were calculated for FVIII-SQ amplification in each reaction by the Light Cycler 480 Software v1.5.1. using Abs quant/2nd derivative max analysis (Roche). An 8‑point standard curve was prepared using known amounts of a linearized, purified hFVIII-SQ DNA fragment diluted in genomic DNA extracted from untreated mouse liver tissue used as a matrix. The average Cp value for each sample was interpolated to the standard curves, giving copies of FVIII-SQ transgene per reaction, which were then converted to copies per liver.

## Immunohistochemistry

Slides were baked, deparaffinized, and rehydrated. Antigen retrieval solution Bond Epitope Retrieval 1 was used (Leica Biosystems, Buffalo Grove, IL; catalog no. AR9961). Sections were blocked with Peroxidazed 1 (BioCare Medical, Concord, CA) and Background Punisher (BioCare Medical, Concord, CA). Anti-PDGFRα (EMD Millipore, catalog no. 07-276) was diluted to 1 µg/mL in Ventana reaction buffer (Ventana Medical Systems, Tucson, AZ) and applied at 1-hour intervals for 5 hours. Rabbit on Rodent HRP (BioCare Medical, Concord, CA) secondary antibody was used to detect anti-PDGFRα. Tertiary antibody TSA Plus Cyanine 3 (Akoya Biosciences, Menlo Park, CA) was diluted in TSA buffer (Advanced Cell Diagnostics, Newark, CA) at 1:1500 and applied to the slides as a tertiary step for 10 minutes at room temperature. Slides were subjected to a secondary epitope retrieval step using Bond Epitope Retrieval 1 and incubated at 95°C for 30 minutes. Slides were blocked with Peroxidazed 1 and Background Punisher. Anti–viral protein 3 (VP3) antibody (Novus Biologicals, Centennial, CO) was diluted to 1 µg/mL in Ventana reaction buffer and applied for 1 hour at room temperature. Rabbit on Rodent HRP secondary antibody was used to detect anti-VP3. Tertiary antibody TSA Plus Cyanine 5 (Akoya Biosciences, Menlo Park, CA) was diluted in TSA buffer at 1:1500 and applied to the slides as a tertiary step for 10 minutes at room temperature. Slides were subjected to a tertiary epitope retrieval step using Bond Epitope Retrieval 1 and incubated at 95°C for 30 minutes. Slides were blocked using 8% normal donkey serum, 0.4% bovine serum albumin, and 1.2% Triton X-100 in 1x Tris-buffered saline for 30 minutes at room temperature. Anti–tomato lectin preconjugated with fluorescein isothiocyanate (Sigma-Aldrich, Burlington, MA) was diluted in Ventana reaction buffer to 2 μg/mL and applied at 1-hour intervals for 5 hours at room temperature. Slides were washed, counterstained with 4′,6-diamidino-2-phenylindole, and mounted with Fluoromount G. Slides were imaged on a Zeiss Axio Scan.Z1 using a Plan-Apochromat 20x/0.8 objective equipped with a Hamamatsu Orca Flash camera. One whole section of liver was acquired per animal, and 2 regions were randomly selected for export and image analysis. Total PDGFRα and VP3 hepatocytes were counted with custom macros using Visiopharm v 2020.09 software (Visiopharm A/S, Hørsholm, Denmark).

# Supplemental figures

**Supplemental Figure S1. Effects of prophylactic prednisolone over 12 weeks. A)** Serum hA1AT levels from mice dosed with 6x10^13^ vg/kg AAV5-HLP-hA1AT. **B)** Percent of hepatocytes staining positive for vector DNA. **C)** Levels of overall vector DNA copies/diploid genomes in mouse livers at 12 weeks (n = 7) by ddPCR. **D)** Pearson correlation between vector DNA and transgene RNA, and **E)** Pearson correlation between transgene RNA and protein in mice dosed with AAV5-HLP-hA1AT and prophylactic corticosteroids or water in the 12-week study.

**A)**

**
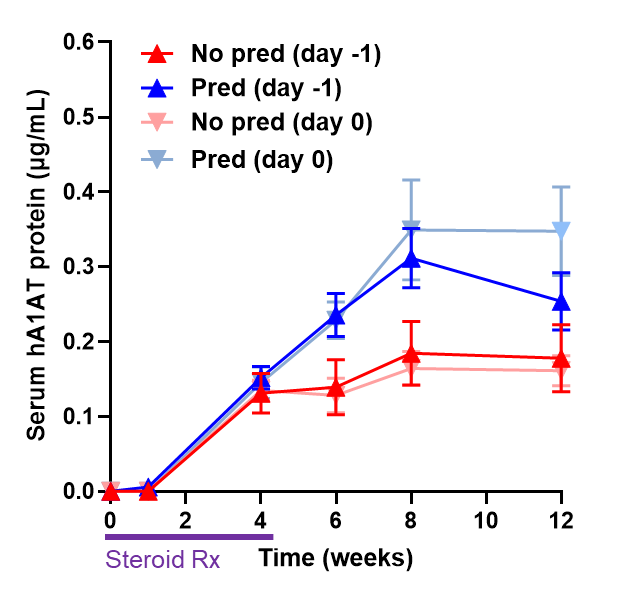
**

**B)
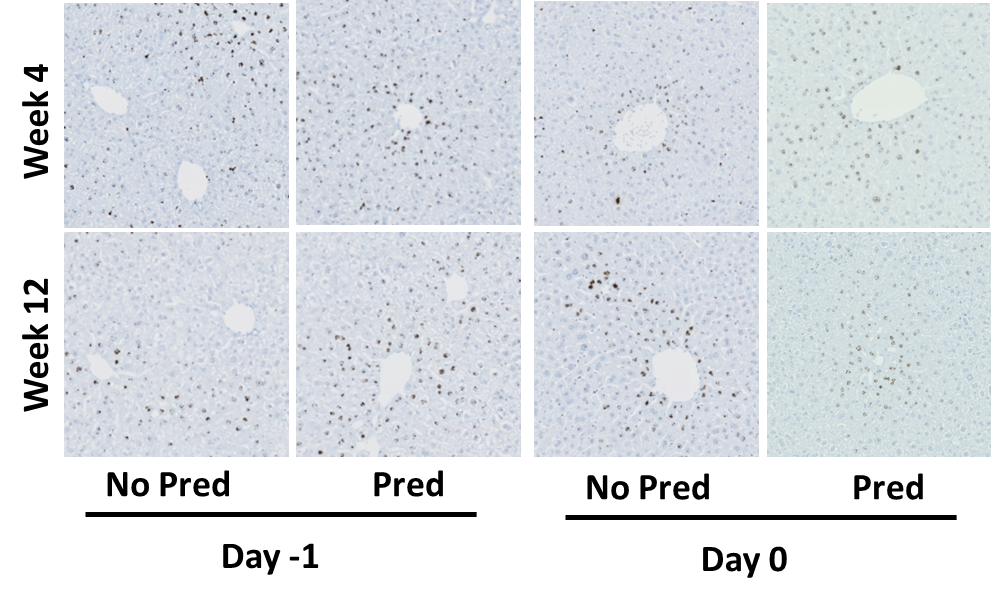
**

**
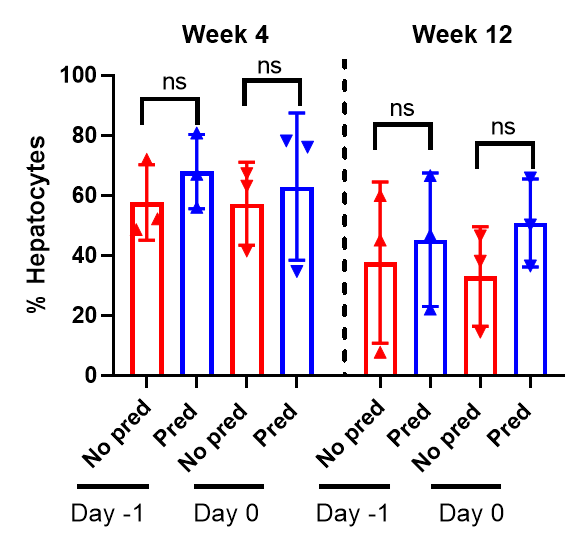
**

**C)**

**
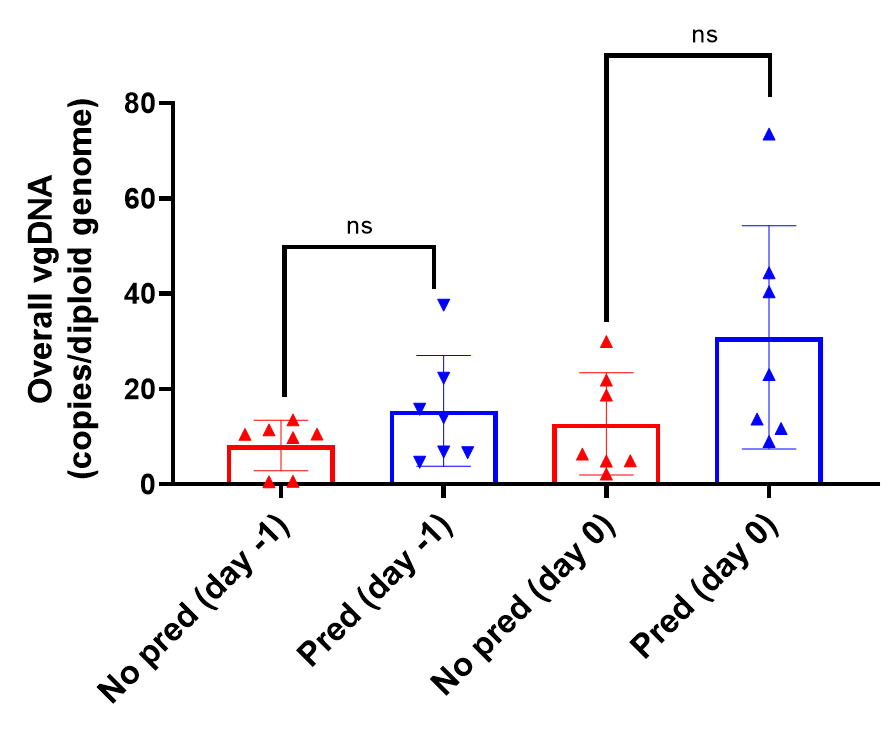
**

**D)**


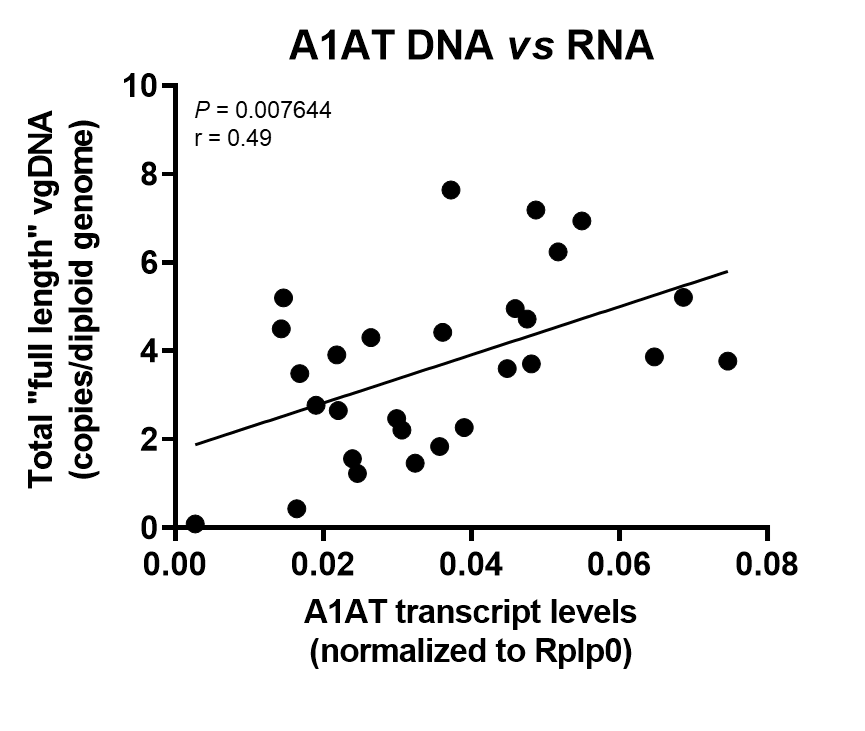


**E)**


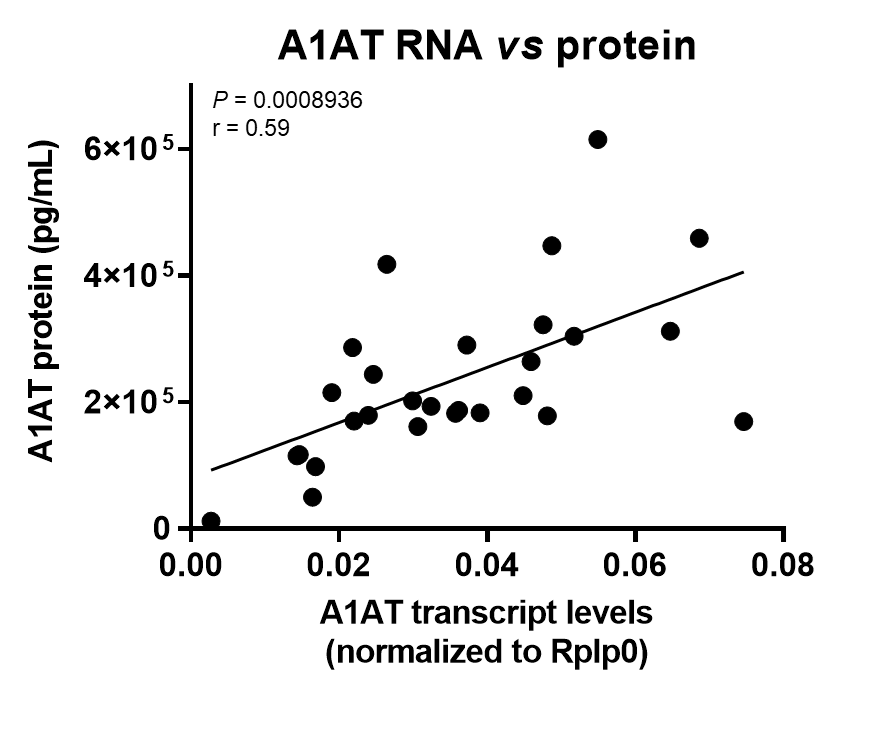


Rplp0 is a housekeeping gene in mice. In panel A, data are mean ± SEM. In panels B and C, data are mean ± SD. In panel B, significance was calculated using an ordinary one-way ANOVA followed by a Tukeys multiple comparisons test. For panel C, a Student’s t-test was performed.

AAV, adeno-associated virus; AAV5-HLP-hA1AT, AAV serotype 5 Hybrid Liver Promoter human A1AT; A1AT, α1-antitrypsin; ddPCR, droplet digital PCR; DNA, deoxyribonucleic acid; h, hour; ns, not significant; pred, prednisolone; RNA, ribonucleic acid; Rplp0, ribosomal protein lateral stalk subunit P0; Rx, treatment; SD, standard deviation; SEM, standard error of the mean; vg, vector genome.

**Supplemental Figure S2. Effect of prophylactic immune suppression treatment in** **non-human primates.** Levels of full-length vector DNA in the liver of cynomolgus monkeys treated with 6x10^13^ vg/kg of AAV5-hFVIII-SQ taken down at week 13 (n = 4, no immune suppression) and week 26 (n = 4, with immune suppression) by drop-phase ddPCR.


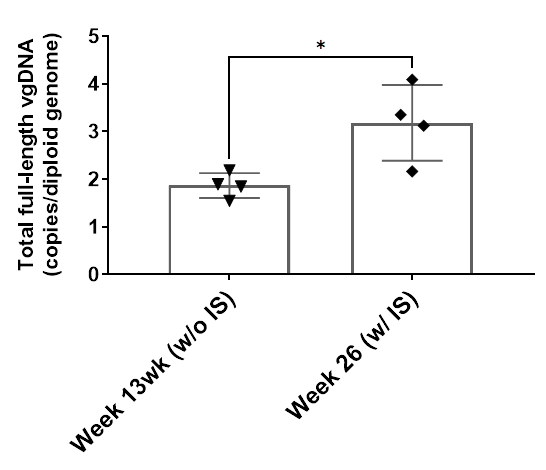


*, *P* <0.05 (t-test). AAV5-hFVIII-SQ, valoctocogene roxaparvovec; DNA, deoxyribonucleic acid; ddPCR, droplet digital polymerase chain reaction; IS, immunosuppressants; vg, vector genome.

**Supplemental Figure S3. Effect of prophylactic prednisolone treatment on immune system activation. A)** Normalized IL-1b levels across treatment groups using RNAseq. **B)** Plasma C3b levels at takedown by ELISA.

**A)**

**
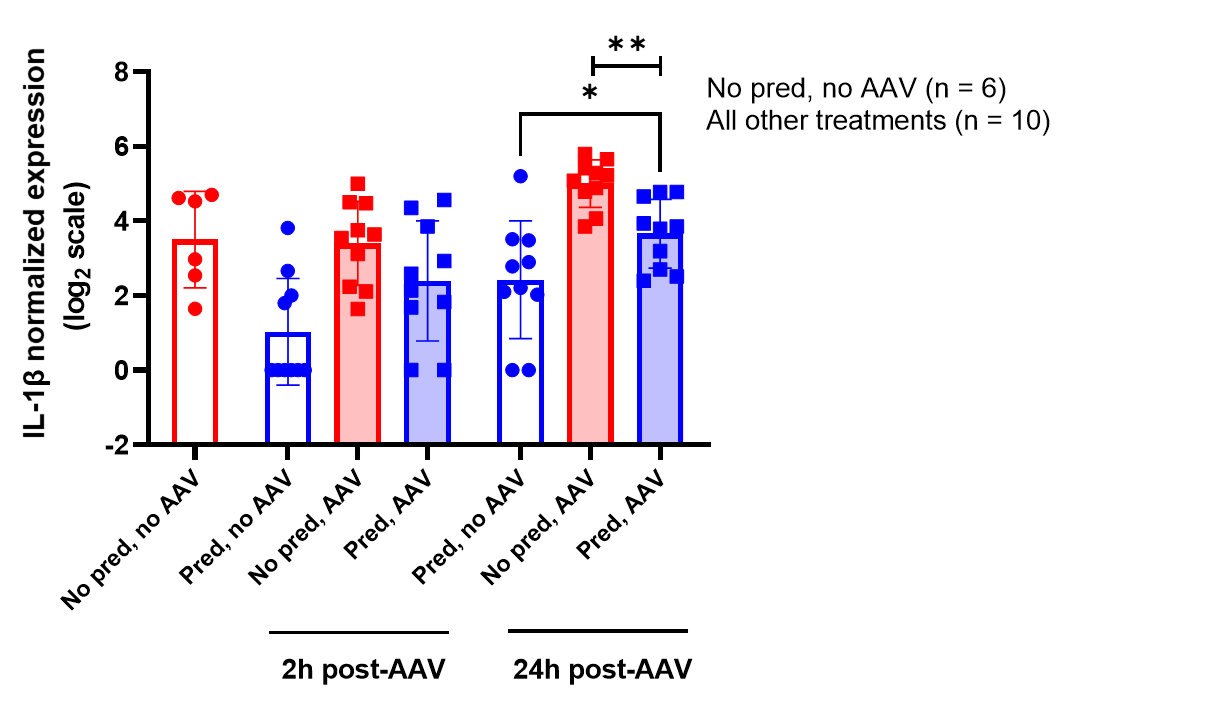
**

**B)**


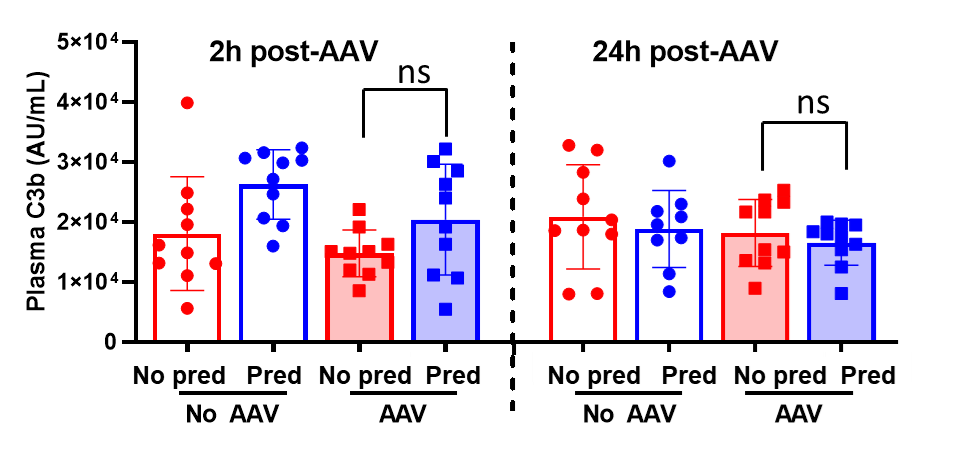


ns, not significant; **P* <0.05; ***P* <0.005.

Bar graphs show mean ± SD; symbols are individual data points. AAV denotes AAV5-HLP-hA1AT dosing. In panel A, a one-way ANOVA showed no significant differences where significance is not indicated. For panel B, a Welch’s unpaired t-test was performed.

AAV, adeno-associated virus; AAV5-HLP-hA1AT, AAV serotype 5 Hybrid Liver Promoter hA1AT; ANOVA, analysis of variance; C3b, complement component 3b; ELISA, enzyme-linked immunoassay; h, hour; hA1AT, human α1-antitrypsin; IL-1b, interleukin-1b; pred, prednisolone; SD, standard deviation.

**Supplemental Figure S4. Plasma ALT concentration in terminal samples.** Samples were taken from mice **A)** 2 and 24 hours post-AAV dosing and **B)** over 12 weeks post-AAV dosing.

**A)**


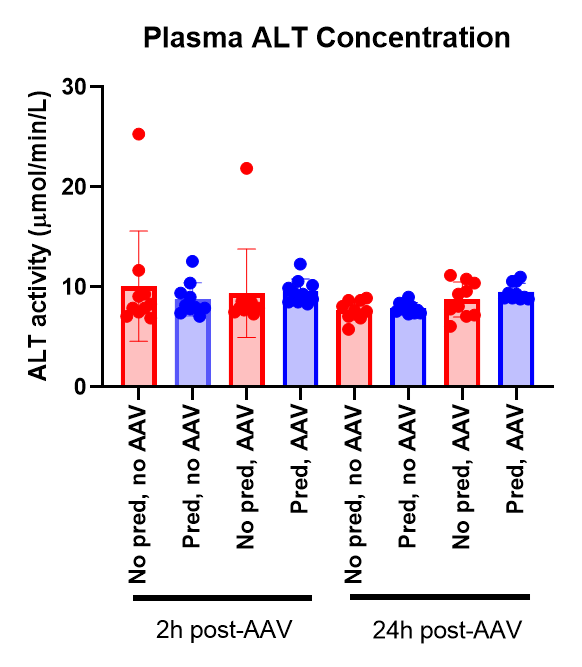


**B)**


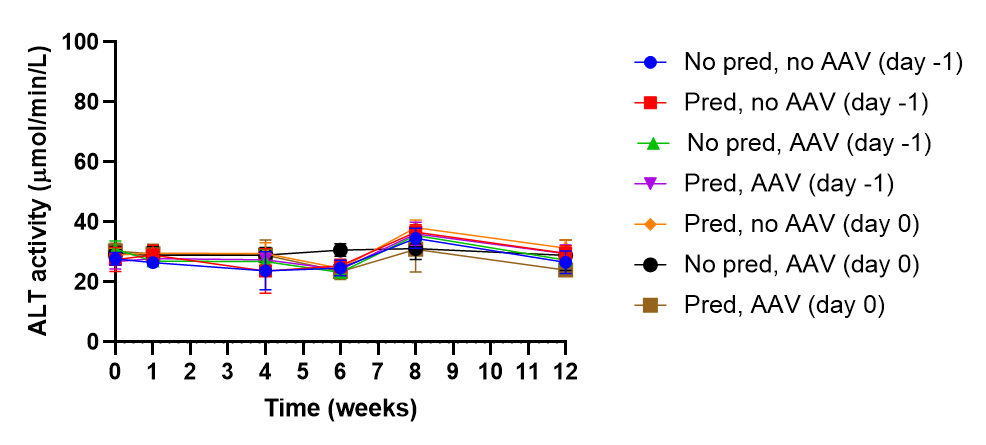


Data are mean ± SD; symbols are individual data points. AAV denotes AAV5-HLP-hA1AT dosing. The normal range of ALT in mice is 18–94 U/L.^5^ In panel B, mice were dosed with prednisolone (pred) or water (no pred) either 2 or 24 hours before dosing with AAV.

AAV, adeno-associated virus; AAV5-HLP-hA1AT, AAV serotype 5 Hybrid Liver Promoter human α1-antitrypsin; ALT, alanine aminotransferase; h, hour; pred, prednisolone; SD, standard deviation.

**Supplemental Figure S5. Effects of prednisolone and AAV5 treatment on the N-glycome of murine liver.**


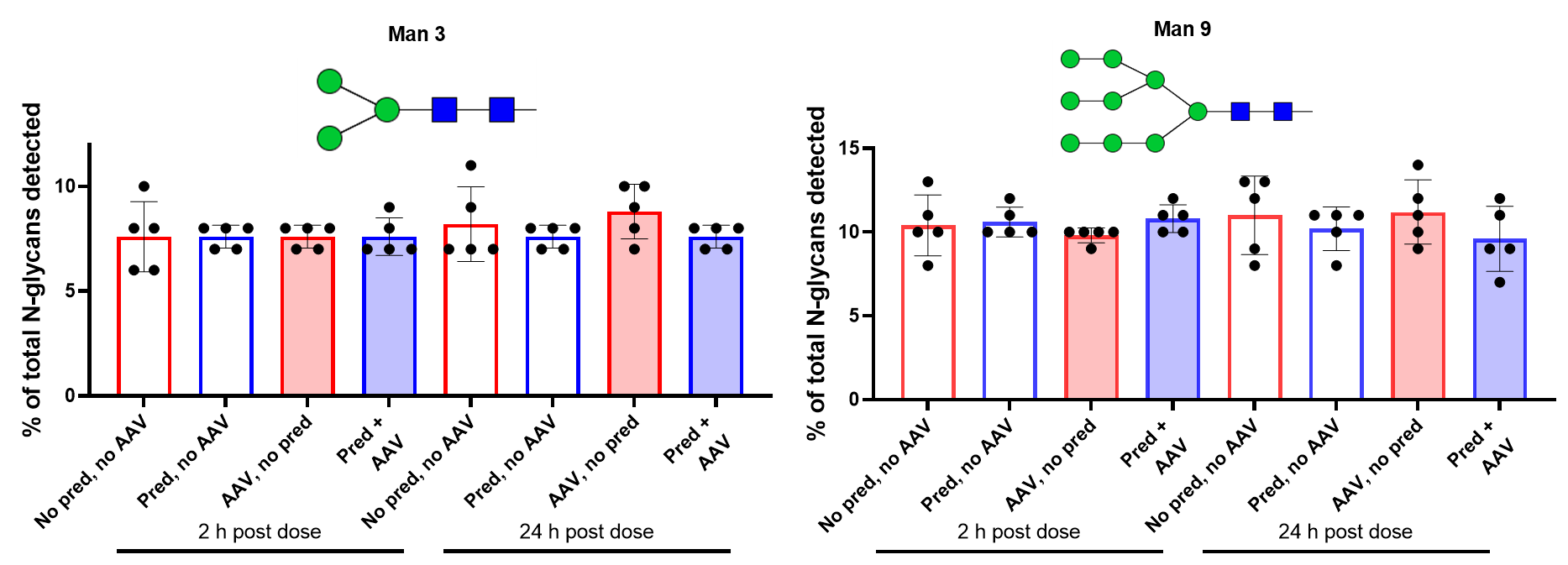


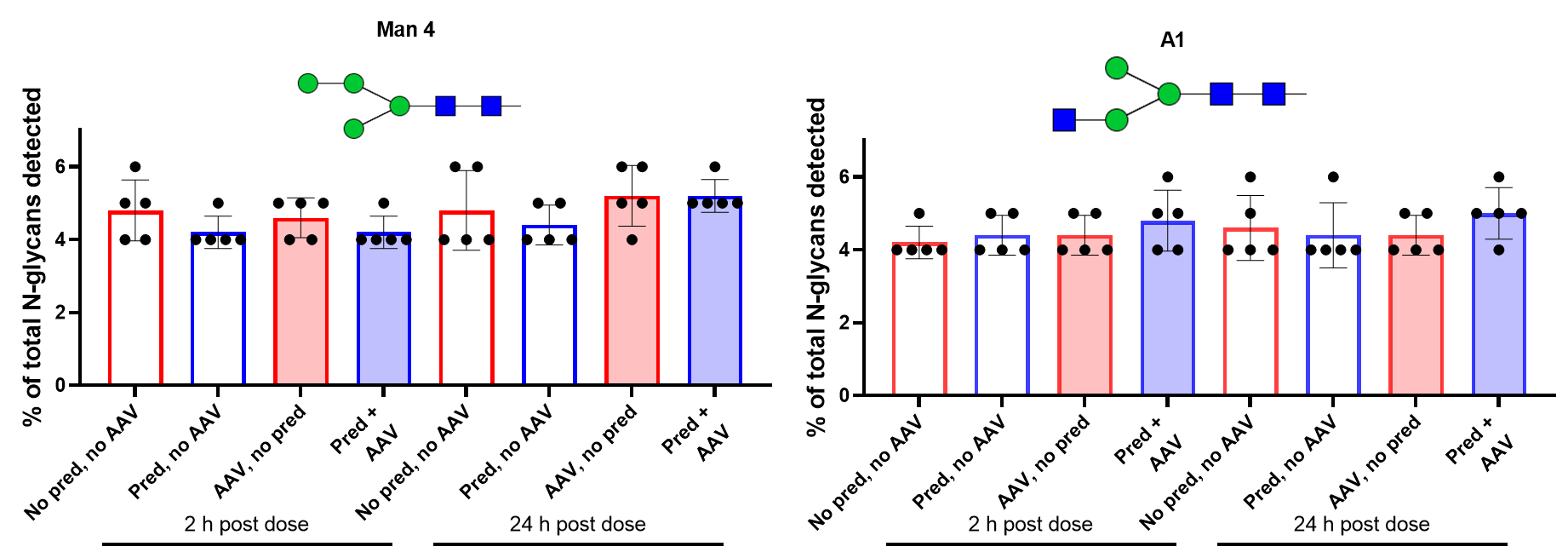


**
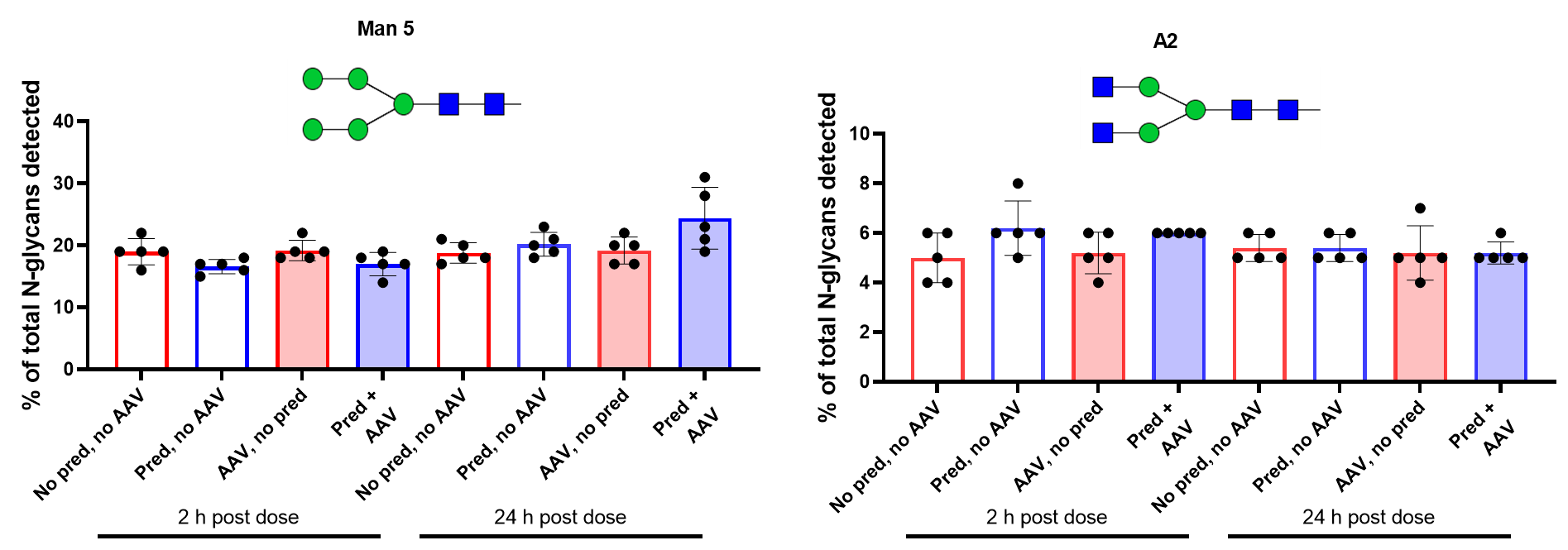
**


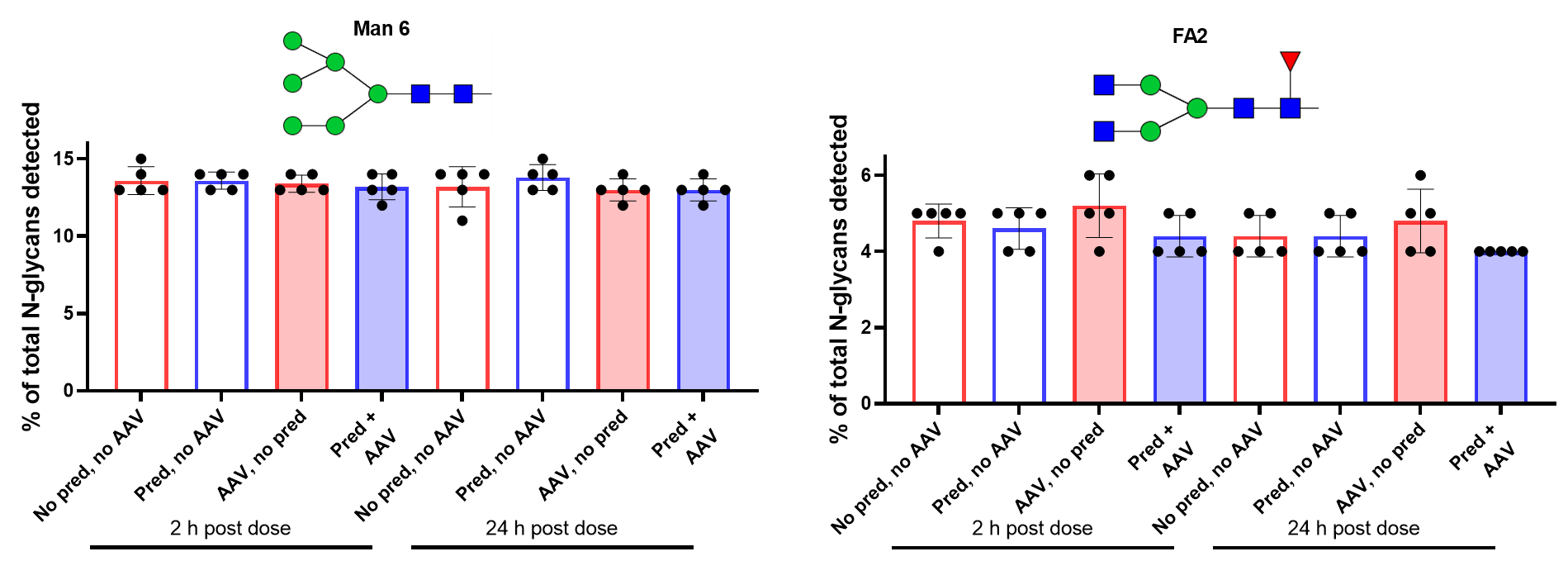


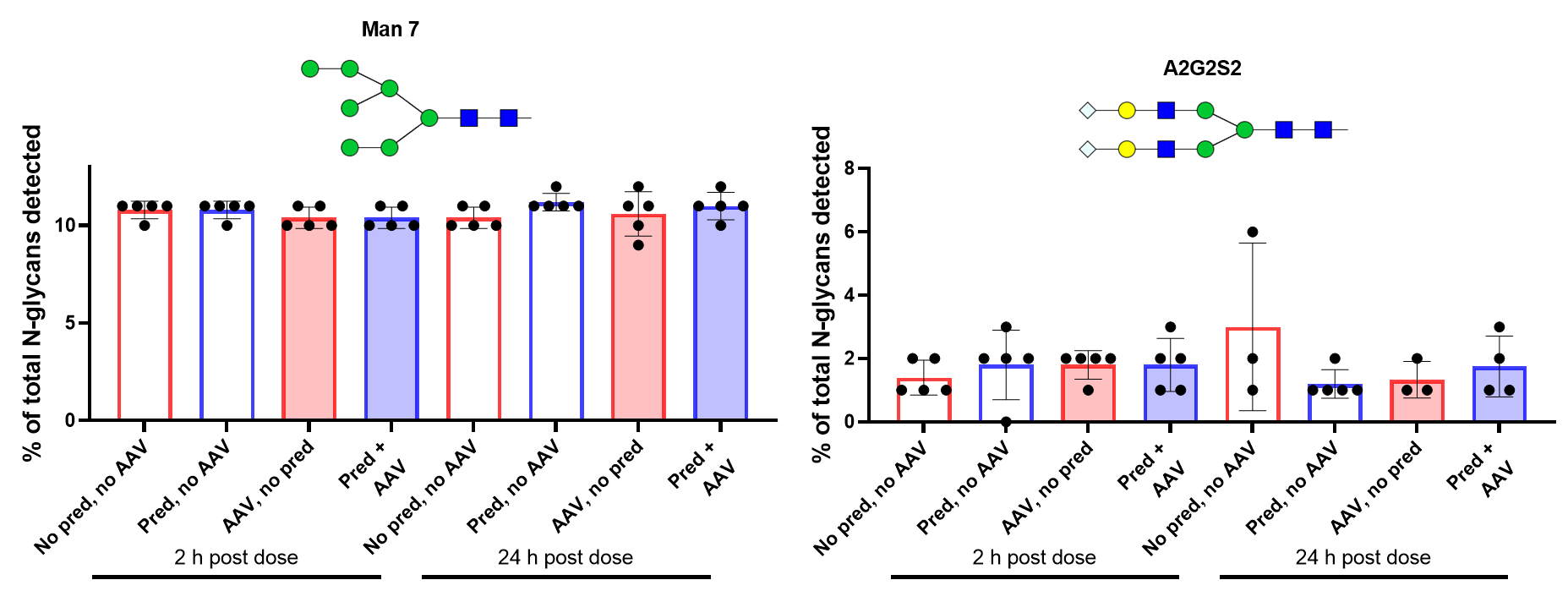


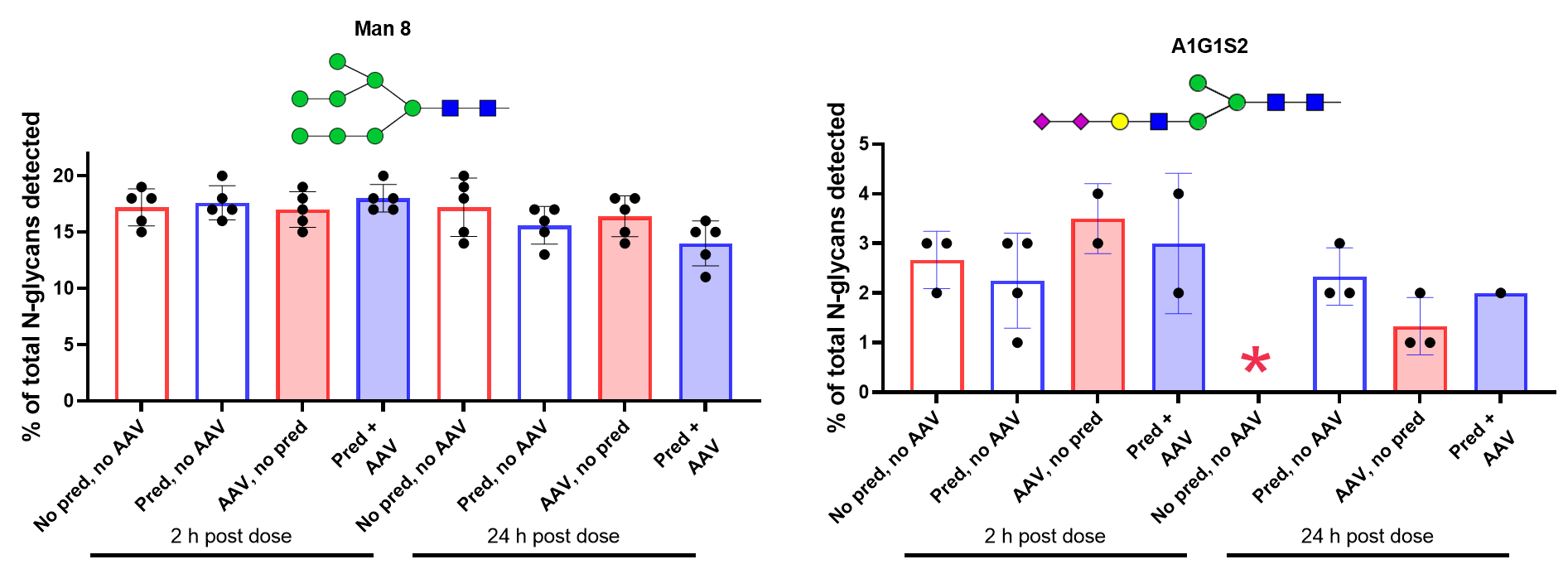
Levels of high mannose, asialo, and sialo N-glycans were analyzed in mice livers from 4 treatments groups: NT, prednisolone-treated, AAV5-treated, and prednisolone + AAV5-treated at 2 time points post treatment (2 hours and 1 day). All N-glycans are reported as a percent of the total ion intensity of all the N-glycans detected for an animal. Groups marked with * signify that this glycan species was below the limit of detection for all animals within this group. Putative glycan structures were determined by mass spectrometry. AAV denotes vector AAV5-HLP-hA1AT.

AAV, adeno-associated virus; AAV5-HLP-hA1AT, AAV serotype 5 Hybrid Liver Promoter human α1-antitrypsin; h, hour; NT, no treatment; pred, prednisolone. Statistical analysis was performed using unpaired t-test.

**Supplemental Figure S6.** **Bulk human tissue gene expression for PDGFRα in the liver.**

**
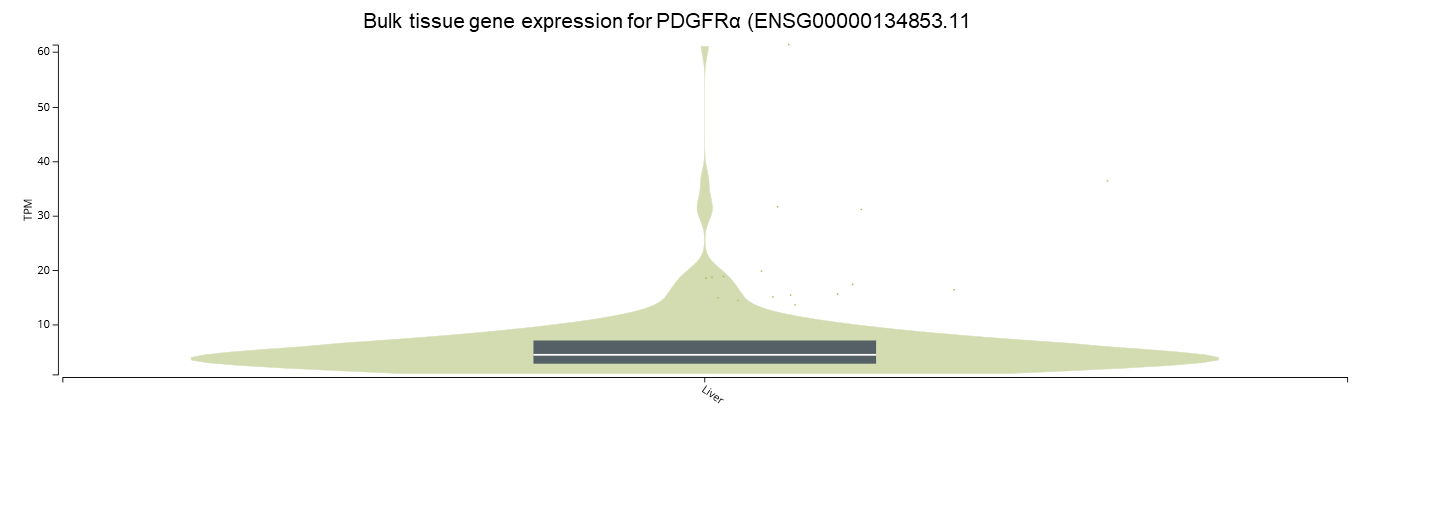
**

N = 226. Data are from the Genotype-Tissue Expression Project, supported by the Common Fund of the Office of the Director of the National Institutes of Health, and by the National Cancer Institute; National Human Genome Research Institute; National Heart, Lung, and Blood Institute; National Institute on Drug Abuse; National Institute of Mental Health; and National Institute of Neurological Disorders and Stroke. These data were obtained from database of Genotypes and Phenotypes (dbGaP) accession number phs000424.v8.p2 on March 11, 2022 at <https://gtexportal.org/home/gene/PDGFRA>.

PDGFRα, platelet-derived growth factor receptor alpha; TMP, transcripts per million.

# Supplemental tables

**Supplemental Table S1. Differential gene expression due to prophylactic prednisolone treatment and AAV5-HLP-A1AT treatment (time after dosing).**

| Time post-AAV dose | % change between treatments | No pred/no AAV vs pred/no AAV | No pred/AAV vs  No pred/no AAV | Pred/AAV vs  No pred/AAV |
| --- | --- | --- | --- | --- |
| 2 h | >25 | 4372 genes | 717 genes | 3793 genes |
|  | >50 | 2954 genes | 57 genes | 2432 genes |
| 24 h | >25 | 1162 genes | 410 genes | 1599 genes |
|  | >50 | 218 genes | 8 genes | 118 genes |

Pred treatment was 2 hours before AAV dosing. AAV treatment was with 6x10^13^ vg/kg AAV5-HLP-A1AT. AAV denotes vector AAV5-HLP-hA1AT.

AAV, adeno-associated virus; AAV5-HLP-hA1AT, AAV serotype 5 Hybrid Liver Promoter human α1-antitrypsin; h, hour; pred, prednisolone.

**Supplemental Table S2. Effect of prednisolone at 2 h using** **Olink protein expression analysis.**

| Assay | UniProt | NPX difference | Pred + AAV | No pred + AAV | *P* value | Adj. *P* value |
| --- | --- | --- | --- | --- | --- | --- |
| Ccl3 | P10855 | –0.65 | 1.87 | 2.52 | 4E–07 | 3E–05 |
| IL-5 | P04401 | –1.73 | 0.83 | 2.56 | 7E–05 | 0.0030 |
| Tpp1 | O89023 | 0.33 | 6.92 | 6.59 | 0.0005 | 0.0130 |
| Wfikkn2 | Q7TQN3 | –0.35 | 4.52 | 4.87 | 0.0007 | 0.0130 |
| Tnfrsf11b | O08712 | –0.71 | 4.77 | 5.47 | 0.0007 | 0.0130 |
| Gfra1 | P97785 | 0.41 | 3.71 | 3.30 | 0.0010 | 0.0150 |
| Clmp | Q8R373 | –0.32 | 6.37 | 6.69 | 0.0013 | 0.0162 |
| Wisp1 | O54775 | –0.35 | 5.03 | 5.37 | 0.0014 | 0.0162 |
| Dll1 | Q61483 | –0.40 | 3.81 | 4.22 | 0.0024 | 0.0245 |
| Ccl20 | O89093 | 1.14 | 8.76 | 7.61 | 0.0030 | 0.0269 |
| Tnfsf12 | O54907 | –0.37 | 3.12 | 3.49 | 0.0032 | 0.0269 |
| Lgmn | O89017 | –0.33 | 4.78 | 5.11 | 0.0040 | 0.0310 |
| Ccl2 | P10148 | –1.23 | 7.85 | 9.08 | 0.0046 | 0.0326 |
| Csf2 | P01587 | –0.29 | 0.11 | 0.40 | 0.0195 | 0.1282 |
| Erbb4 | Q61527 | 0.30 | 5.32 | 5.02 | 0.0235 | 0.1441 |
| Hgf | Q08048 | –0.37 | 3.80 | 4.17 | 0.0432 | 0.2484 |

Mean differences in NPX (log_2_ scale) between pred + AAV and no pred + AAV-treated mice at 2 hours post-dose; n = 10/group. *P* values are from t-tests performed on NPX values and adjusted for multiple testing using the Benjamini-Hochberg procedure in Olink Insights Stat Analysis. Cutoff for inclusion was *P* value <0.05. Adj. *P* value denotes adjusted *P* value. AAV denotes vector AAV5-HLP-hA1AT. (<https://olinkproteomics.shinyapps.io/OlinkInsightsStatAnalysis/>)

AAV, adeno-associated virus; AAV5-HLP-hA1AT, AAV serotype 5 Hybrid Liver Promoter human α1-antitrypsin; adj, adjusted; E, exponential; h, hour; NPX, normalized protein expression; pred, prednisolone.

**Supplemental Table S3. Effect of prednisolone at 24 h using Olink protein expression analysis.**

| Assay | UniProt | NPX difference | Pred + AAV | No pred + AAV | *P* value | Adj. *P* value |
| --- | --- | --- | --- | --- | --- | --- |
| Parp1 | P11103 | 0.92 | 4.54 | 3.62 | 0.0021 | 0.1891 |
| Vsig2 | Q9Z109 | –0.31 | 3.13 | 3.44 | 0.0075 | 0.3055 |
| Ntf3 | P20181 | 0.22 | 1.62 | 1.40 | 0.0161 | 0.3055 |
| Il17a | Q62386 | 0.89 | 0.93 | 0.03 | 0.0174 | 0.3055 |
| Fst | P47931 | 0.29 | 8.12 | 7.82 | 0.0205 | 0.3055 |
| Vegfd | P97946 | –0.17 | 3.41 | 3.58 | 0.0205 | 0.3055 |
| Pak4 | Q8BTW9 | 0.33 | 0.07 | –0.25 | 0.0232 | 0.3055 |
| Tgfbr3 | O88393 | –0.15 | 3.80 | 3.95 | 0.0312 | 0.3586 |
| Tnr | Q8BYI9 | –0.16 | 3.28 | 3.43 | 0.0391 | 0.3995 |

Mean differences in NPX (log_2_ scale) between pred + AAV and no pred + AAV-treated mice at 2 hours post-dose; n = 10/group. *P* values are from t-tests performed on NPX values and adjusted for multiple testing using the Benjamini-Hochberg procedure in Olink Insights Stat Analysis. Cutoff for inclusion was *P* value <0.05. Adj. *P* value denotes adjusted *P* value. AAV denotes vector AAV5-HLP-hA1AT. (<https://olinkproteomics.shinyapps.io/OlinkInsightsStatAnalysis/>)

AAV, adeno-associated virus; AAV5-HLP-hA1AT, AAV serotype 5 Hybrid Liver Promoter human α1-antitrypsin; adj, adjusted; h, hour; NPX, normalized protein expression; pred, prednisolone.

**Supplemental Table S4. Effect of AAV at2 h using Olink protein expression analysis.**

| Assay | UniProt | NPX difference | Pred + AAV | Pred + no AAV | *P* value | Adj. *P* value |
| --- | --- | --- | --- | --- | --- | --- |
| Gfra1 | P97785 | 0.57 | 3.71 | 3.13 | 0.0001 | 0.0095 |
| Tnfrsf12a | Q9CR75 | 0.50 | 3.40 | 2.90 | 0.0002 | 0.0099 |
| S100a4 | P07091 | 0.43 | 5.72 | 5.29 | 0.0018 | 0.0433 |
| Plxna4 | Q80UG2 | –0.94 | 2.30 | 3.24 | 0.0020 | 0.0433 |
| Fstl3 | Q9EQC7 | 0.29 | 5.95 | 5.66 | 0.0024 | 0.0433 |
| Ghrl | Q9EQX0 | 0.44 | 3.39 | 2.95 | 0.0135 | 0.2065 |
| Rgma | Q6PCX7 | 0.12 | 2.75 | 2.62 | 0.0176 | 0.2320 |
| Fas | P25446 | 0.24 | 4.14 | 3.90 | 0.0247 | 0.2745 |
| Csf2 | P01587 | 0.23 | 0.11 | –0.12 | 0.0275 | 0.2745 |
| Ccl2 | P10148 | 0.85 | 7.85 | 7.00 | 0.0298 | 0.2745 |
| Il1b | P10749 | 0.24 | 1.32 | 1.07 | 0.0359 | 0.3001 |
| Lgmn | O89017 | 0.26 | 4.78 | 4.52 | 0.0410 | 0.3140 |
| Mia | Q61865 | 0.21 | 5.41 | 5.21 | 0.0526 | 0.3199 |
| Tnfrsf11b | O08712 | 0.32 | 4.77 | 4.45 | 0.0540 | 0.3199 |

Mean differences in NPX (log_2_ scale) between pred + AAV and pred + no AAV-treated mice at 2 hours post-dose; n = 10/group. *P* values are from t-tests performed on NPX values and adjusted for multiple testing using the Benjamini-Hochberg procedure in Olink Insights Stat Analysis. Cutoff for inclusion was *P* value <0.05. Adj. *P* value denotes adjusted *P* value. AAV denotes vector AAV5-HLP-hA1AT. (<https://olinkproteomics.shinyapps.io/OlinkInsightsStatAnalysis/>).

AAV, adeno-associated virus; AAV5-HLP-hA1AT, AAV serotype 5 Hybrid Liver Promoter human α1-antitrypsin; adj, adjusted; h, hour; NPX, normalized protein expression; pred, prednisolone.

**Supplemental Table S5. Effect of AAV at 24 h using Olink protein expression analysis.**

| Assay | UniProt | NPX  difference | Pred +  AAV | Pred +  no AAV | *P* value | Adj. *P* value |
| --- | --- | --- | --- | --- | --- | --- |
| Epo | P07321 | 1.27 | 5.56 | 4.29 | 0.0006 | 0.0548 |
| Il17f | Q7TNI7 | 0.54 | 1.00 | 0.45 | 0.0018 | 0.0819 |
| Tnr | Q8BYI9 | –0.18 | 3.28 | 3.46 | 0.0070 | 0.1557 |
| Il17a | Q62386 | 1.03 | 0.93 | –0.11 | 0.0082 | 0.1557 |
| Adam23 | Q9R1V7 | 0.17 | 1.14 | 0.97 | 0.0092 | 0.1557 |
| Fstl3 | Q9EQC7 | 0.18 | 5.84 | 5.67 | 0.0102 | 0.1557 |
| Il1b | P10749 | 0.34 | 1.68 | 1.34 | 0.0214 | 0.2681 |
| Ccl20 | O89093 | 0.48 | 7.21 | 6.73 | 0.0233 | 0.2681 |
| Dll1 | Q61483 | 0.17 | 3.99 | 3.81 | 0.0388 | 0.3288 |
| Kitlg | P20826 | 0.21 | 0.46 | 0.25 | 0.0392 | 0.3288 |
| Cyr61 | P18406 | –0.74 | 4.92 | 5.65 | 0.0393 | 0.3288 |
| Fas | P25446 | 0.15 | 3.90 | 3.75 | 0.0471 | 0.3436 |
| Dlk1 | Q09163 | 0.19 | 3.14 | 2.95 | 0.0486 | 0.3436 |

Mean differences in NPX (log_2_ scale) between pred + AAV and pred + no AAV-treated mice at 2 hours post-dose; n = 10/group. *P* values are from t-tests performed on NPX values and adjusted for multiple testing using the Benjamini-Hochberg procedure in Olink Insights Stat Analysis. Cutoff for inclusion was *P* value <0.05. Adj. *P* value denotes adjusted *P* value. AAV denotes vector AAV5-HLP-hA1AT. (<https://olinkproteomics.shinyapps.io/OlinkInsightsStatAnalysis/>)

AAV, adeno-associated virus; AAV5-HLP-hA1AT, AAV serotype 5 Hybrid Liver Promoter human α1-antitrypsin; adj, adjusted; h, hour; NPX, normalized protein expression; pred, prednisolone.

**Supplemental Table S6. Hallmark enrichment analysis showing complement pathway genes significantly upregulated at 2 h on comparing prednisolone + AAV vs prednisolone + no AAV.**

| **Gene symbol** | **log_2_FC** | ***P* value** | ***P*-adj** | **Key function/complement pathway** |
| --- | --- | --- | --- | --- |
| Cxcl1 | 3.11 | 1.58E-07 | 2.24E-04 | Inflammation |
| S100a9 | 2.65 | 3.01E-04 | 1.64E-02 | Antimicrobial activity |
| Ppp2cb | 0.29 | 1.62E-03 | 3.58E-02 | Akt/TLR4 signaling |
| Ctso | 0.30 | 2.01E-03 | 3.96E-02 | Class I MHC Ag proc |
| Irf1 | 0.52 | 2.70E-03 | 4.54E-02 | IFN-γ signaling |
| Lipa | 0.30 | 2.73E-03 | 4.56E-02 | Cholesterol transport |
| Vcpip1 | 0.26 | 3.16E-03 | 4.92E-02 | Deubiquitination |
| Cfb | 1.09 | 1.14E-02 | 9.89E-02 | Complement factor B |
| C9 | 0.47 | 1.88E-02 | 1.29E-01 | MAC forms lytic pore by multiple C9 molecules |

AAV, adeno-associated virus; adj, adjusted; E, exponential; h, hour; IFN, interferon; MAC, membrane attack complex; MHC, major histocompatibility; TLR4, toll-like receptor 4.

# Supplemental references

1. Sihn CR, Handyside B, Liu S, et al. Molecular analysis of AAV5-hFVIII-SQ vector-genome-processing kinetics in transduced mouse and nonhuman primate livers. Mol Ther Methods Clin Dev 2022;24:142-153.

2. Skeene K, Walker M, Clarke G, et al. One Filter, one sample, and the N- and O-Glyco(proteo)me: toward a system to study disorders of protein glycosylation. Anal Chem 2017;89:5840-5849.

3. Ceroni A, Maass K, Geyer H, et al. GlycoWorkbench: a tool for the computer-assisted annotation of mass spectra of glycans. J Proteome Res 2008;7:1650-1659.

4. Steering Committee. Consortium for functional Glycomics. Available from: <http://www.functionalglycomics.org/> [Last accessed March 8, 2022]. 2011.

5. Otto GP, Rathkolb B, Oestereicher MA, et al. Clinical chemistry reference intervals for C57BL/6J, C57BL/6N, and C3HeB/FeJ mice (Mus musculus). J Am Assoc Lab Anim Sci 2016;55:375-386.
